# Macrophage-Orchestrated Metabolic Sensing Drives IBD Pathogenesis: A Framework for Targeted Therapy

**DOI:** 10.7150/ijbs.131531

**Published:** 2026-05-25

**Authors:** Guangqi Guan, Xuelin Sun, Yaoxing Chen, Qingyun Guo, Yulan Dong

**Affiliations:** 1 College of Veterinary Medicine, China Agricultural University, Beijing, People's Republic of China.; 2 Department of Pharmacy, Beijing Hospital, National Center of Gerontology; Institute of Geriatric Medicine, Chinese Academy of Medical Sciences, Beijing, 100730, People's Republic of China.; 3 Milu Conservation Research Unit, Beijing Milu Ecological Research Center, Beijing 100076, People's Republic of China.

**Keywords:** intestinal macrophages, immunometabolism, microbiota-derived metabolites, inflammatory bowel disease, conceptual framework

## Abstract

Inflammatory bowel disease (IBD) arises from dysregulated interactions among the gut microbiota, immune system, and intestinal epithelium. Intestinal macrophages are central to these processes, yet are often viewed primarily as downstream inflammatory effectors. Here, we present a conceptual review that reframes intestinal macrophages as metabolic sensors and regulatory hubs that orchestrate inflammatory persistence or resolution. We propose a Macrophage-Orchestrated Metabolic Sensor (MOMS) framework organized into three coordinated layers: Sense, in which macrophages detect microbial- and host-derived metabolites; Switch, in which metabolic and epigenetic reprogramming stabilizes intracellular inflammatory or reparative states; and Command, in which these stabilized states drive epithelial repair, immune-cell recruitment, or fibrotic remodeling. Integrating evidence from immunometabolism, microbiome research, and single-cell biology, we identify key molecular nodes—including METTL3 and NLRP3—as programmable regulators of macrophage fate. The MOMS framework generates testable predictions linking macrophage metabolic states to disease severity and treatment responsiveness, and provides a conceptual foundation for precision macrophage-directed therapies in IBD and related immune-metabolic disorders.

## Introduction

Inflammatory bowel disease (IBD), encompassing ulcerative colitis and Crohn's disease, is a chronic and relapsing disorder characterized by dysregulated mucosal immunity and disrupted intestinal homeostasis 1. Despite extensive investigation into adaptive immune responses and epithelial barrier dysfunction, accumulating evidence indicates that intestinal macrophages occupy a central and upstream position in orchestrating both inflammatory activation and tissue repair within the gut 2. Far from being mere scavengers or terminal effectors, intestinal macrophages integrate microbial, metabolic, and environmental cues to actively shape the intestinal immune microenvironment 3.

Recent advances in single-cell transcriptomics, spatial biology, and metabolomics have fundamentally expanded our understanding of macrophage diversity in the intestine, revealing pronounced functional, metabolic, and spatial heterogeneity among macrophage populations 4, 5. These cells exhibit dynamic plasticity along the crypt-luminal axis and respond sensitively to microbiota-derived metabolites, including short-chain fatty acids, indoles, and polyamines, which influence their polarization and persistence 6. Concomitantly, metabolic and epigenetic reprogramming—ranging from shifts between glycolysis and mitochondrial oxidative metabolism to RNA methylation and chromatin remodeling—has emerged as a decisive determinant of macrophage inflammatory behavior and effector function in the inflamed gut 7, 8.

**Figure 1:**
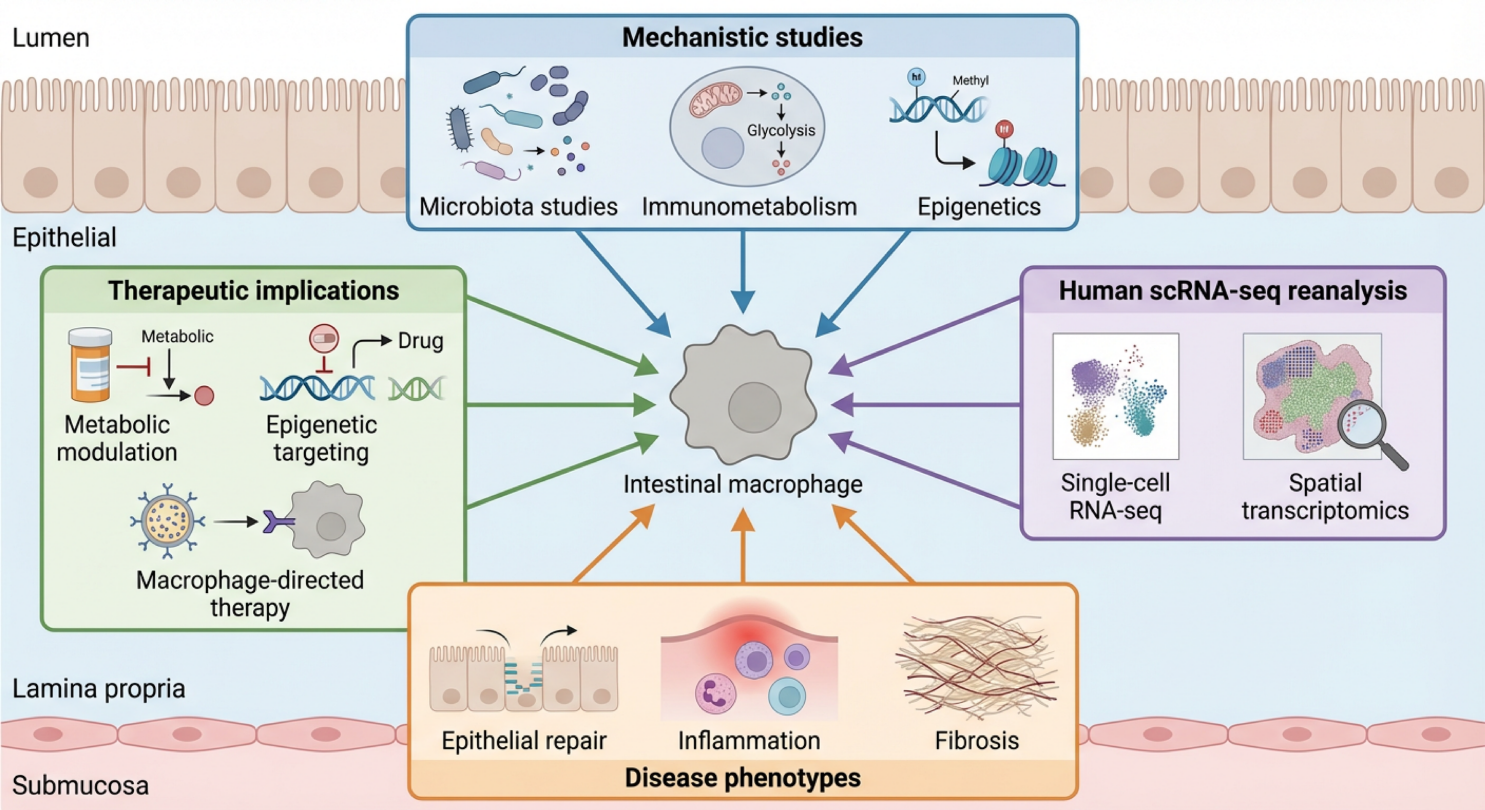
** Multilayered evidence supporting macrophage-centered metabolic decision-making in IBD.** Schematic illustration summarizing converging lines of evidence that motivate the Macrophage-Orchestrated Metabolic Sensor (MOMS) framework. Mechanistic studies of microbiota-derived metabolites, immunometabolic regulation, and epigenetic remodeling converge on intestinal macrophages as central integrators of inflammatory logic. Independent support from human single-cell transcriptomic and spatial profiling highlights disease-associated macrophage expansion and metabolic reprogramming. Together, these data link macrophage-centered sensing and intracellular state transitions to epithelial dysfunction, immune-cell recruitment, fibrotic remodeling, and emerging therapeutic strategies in inflammatory bowel disease.

Although intestinal macrophages are increasingly recognized as key regulators of IBD pathogenesis, existing studies and reviews tend to address macrophage metabolism, microbiota-immune interactions, or inflammatory signaling as largely independent processes. What remains lacking is a unifying conceptual framework that explains how intestinal macrophages integrate diverse metabolic inputs, convert them into stable intracellular decision states, and issue coordinated tissue-level instructions that determine inflammatory persistence or resolution. **Figure 1** summarizes the diverse experimental, omics-based, and translational evidence that collectively supports intestinal macrophages as central integrators of metabolic and inflammatory information in IBD.

Over the past decade, immunometabolism has emerged as a central paradigm for understanding macrophage function in inflammatory disease. Prevailing models largely conceptualize metabolic reprogramming as a downstream consequence of activation, in which shifts toward glycolysis or oxidative phosphorylation provide bioenergetic and biosynthetic support for predefined inflammatory or reparative phenotypes. In this view, metabolic pathways are primarily permissive or supportive modules that sustain macrophage polarization programs initiated by cytokine or pattern-recognition receptor signaling.

While this framework has generated substantial mechanistic insight, it presents several conceptual limitations when applied to chronic inflammatory bowel disease (IBD). First, it assumes a largely linear input-output relationship between environmental stimuli and macrophage activation states, without explicitly accounting for intracellular mechanisms that stabilize or “lock in” these states. Second, it treats metabolic signatures as correlates of polarization rather than determinants of fate, thereby offering limited explanation for the persistence of inflammatory macrophage programs despite cytokine blockade. Third, existing models do not sufficiently explain the spatial and functional heterogeneity of macrophage subsets revealed by single-cell and spatial transcriptomic studies in human IBD, nor do they readily account for variable therapeutic responsiveness across patients.

We propose that metabolism is not merely a downstream consequence of macrophage activation. Instead, metabolic pathways act as an active regulatory layer that shapes macrophage fate. The MOMS framework organizes macrophage behavior into three coordinated layers: sensing environmental metabolites (Sense), stabilizing intracellular programs through metabolic and epigenetic mechanisms (Switch), and executing tissue-level outputs (Command). In this model, environmental cues are actively interpreted through specific receptors and metabolic sensors. These signals are then translated into relatively stable intracellular states that determine whether macrophages promote inflammation or tissue repair. By linking metabolic reprogramming to durable functional outcomes, this framework positions metabolism as a causal driver of disease progression rather than a secondary feature of activation.

Although significant progress has been made in immunometabolism and microbiota-immune interactions, current models do not fully explain how intestinal macrophages integrate diverse metabolic cues into stable functional states. Most frameworks describe metabolic reprogramming as a consequence of cytokine signaling or treat microbial metabolites as isolated modulators of inflammation. These views do not sufficiently account for the persistence of inflammatory macrophage programs in chronic IBD, nor do they explain why cytokine blockade alone often fails to achieve durable remission. A central gap in these models is the lack of an intermediate regulatory layer that stabilizes macrophage states once activated.

To clarify the conceptual advancement of the MOMS framework, it is important to distinguish it explicitly from prior models of macrophage immunometabolism and microbiota-immune interaction. Classical immunometabolism models primarily describe metabolic reprogramming as a downstream adaptation that supports predefined activation states induced by cytokines or pattern-recognition receptors. Microbiota-immune interaction models, in contrast, emphasize the modulatory role of microbial metabolites but do not specify how these signals are stabilized into durable intracellular decision states. Cytokine-centered cascade models further focus on effector outputs without accounting for intracellular metabolic memory. Key conceptual differences between the MOMS framework and previous models of macrophage immunometabolism are summarized in **Table 1**.

The MOMS framework introduces a hierarchical architecture that incorporates an intermediate stabilization layer (“Switch”), in which metabolic and epigenetic circuits generate semi-stable attractor states. This addition enables mechanistic explanations for three phenomena that remain insufficiently addressed by prior models: (1) persistence of inflammatory macrophage programs despite cytokine blockade, (2) spatial heterogeneity of macrophage states within diseased tissue, and (3) patient-to-patient variability in therapeutic responsiveness. By framing metabolism as a fate-determining and state-stabilizing module rather than a passive consequence of activation, MOMS generates testable predictions linking metabolic configuration to disease durability and treatment resistance.

By integrating mechanistic insights with emerging therapeutic strategies, this conceptual Review outlines a translational roadmap linking macrophage metabolic sensing and molecular switching to precision-targeted intervention. Collectively, the MOMS framework reframes intestinal macrophages not as passive participants in inflammation, but as programmable orchestrators of disease dynamics, highlighting actionable nodes for the development of next-generation macrophage-directed therapies in IBD. Notably, reanalysis of publicly available human IBD single-cell transcriptomic datasets reveals disease-associated expansion and metabolic reprogramming of intestinal macrophage subsets, providing independent human evidence consistent with the hierarchical sensing-switching-command logic proposed in the MOMS framework **(Supplementary Figures S1-S5)**.

## Microbial and Metabolic Sensing by Intestinal Macrophages in IBD

Intestinal macrophages reside at a unique anatomical and functional interface between the host and the external environment 9. Constantly exposed to microbial signals and dietary products, they are equipped with a repertoire of pattern-recognition receptors (PRRs) and metabolite-sensing pathways that enable them to function as frontline metabolic sensors. In the context of inflammatory bowel disease (IBD), where microbial dysbiosis and barrier breakdown prevail, this sensing machinery becomes disproportionately activated, converting environmental perturbations into sustained inflammatory programs 10.

A growing body of evidence has established microbial metabolites as critical inputs into macrophage fate determination. Among them, short-chain fatty acids (SCFAs), particularly butyrate and propionate, are the most well-characterized modulators 11. In homeostasis, SCFAs are produced by commensal bacteria through fermentation of dietary fibers and engage G-protein-coupled receptors such as GPR43 and GPR109A on macrophages, promoting anti-inflammatory signaling and enhancing epithelial repair 12, 13. In IBD, reduced abundance of SCFA-producing bacteria leads to diminished butyrate availability, impairing regulatory macrophage functions and skewing them toward a pro-inflammatory phenotype.

Beyond SCFAs, tryptophan-derived metabolites such as indole, indole-3-aldehyde, and other aryl hydrocarbon receptor (AhR) ligands play a pivotal role in macrophage conditioning. Activation of AhR signaling in intestinal macrophages influences the production of IL-22 and other barrier-protective mediators, linking microbial metabolism directly to epithelial regeneration and mucosal integrity 14. Dysregulation of this pathway in IBD contributes to the breakdown of immune tolerance and amplifies inflammatory cascades.

Polyamines and secondary bile acids represent additional layers of microbial-host metabolic crosstalk. Elevated or depleted levels of these metabolites have been shown to remodel macrophage cytokine profiles, alter phagocytic capacity, and modulate antigen presentation. Together, these findings support the notion that intestinal macrophages continuously sample and interpret a complex landscape of microbiota-derived signals, translating these cues into distinct functional outcomes 15, 16. Representative examples of microbial metabolites, their corresponding receptors or signaling pathways, and their effects on macrophage metabolic programming are summarized in **Table 2**.

### Classes of Microbial Inputs That Shape Macrophage Metabolic Sensing

Intestinal macrophages do not respond to individual microbial species in isolation. Rather, they interpret distinct biochemical classes of microbial-derived signals that encode metabolic information. Within the MOMS framework, these microbial inputs constitute the informational substrate of the Sense layer, biasing downstream switch stabilization toward inflammatory or reparative attractor states.

#### SCFA-Producing Commensals and HDAC-Dependent Stabilization

Short-chain fatty acids (SCFAs), including butyrate and propionate, represent one of the most well-characterized microbial signals shaping macrophage fate. These metabolites are primarily generated by fiber-fermenting commensals such as Faecalibacterium prausnitzii and selected Bacteroides species 22.

Rather than acting merely as metabolic substrates, SCFAs function as epigenetic modulators through inhibition of histone deacetylases (HDACs) and activation of G-protein-coupled receptors such as GPR43 and GPR109A 12, 13. HDAC inhibition increases chromatin accessibility at loci associated with IL-10 production and oxidative metabolism, while simultaneously constraining inflammasome priming 23.

Experimental studies demonstrate that extracellular vesicles derived from F. prausnitzii promote macrophage metabolic reprogramming toward oxidative phosphorylation and attenuate chronic colitis-associated fibrosis 24. These findings suggest that SCFA-producing commensals stabilize macrophages in a reparative metabolic configuration, thereby reducing the probability of inflammatory switch locking.

Within the MOMS hierarchy, SCFA depletion in IBD represents not merely a loss of anti-inflammatory tone but a disruption of sensory input fidelity that predisposes macrophages to glycolytic stabilization.

#### Indole-Producing Bacteria and AhR-Mediated Transcriptional Programming

Tryptophan-derived indole metabolites constitute a second major class of microbial signals. Species within the genera Bifidobacterium and Lactobacillus, as well as specific Bacteroides strains, convert dietary tryptophan into indole derivatives that activate the aryl hydrocarbon receptor (AhR) in intestinal macrophages 25.

AhR functions as a ligand-dependent transcription factor that modulates oxidative metabolism, barrier-supportive cytokine production, and inflammasome sensitivity. Activation of AhR signaling enhances IL-22-associated tissue repair circuits and promotes macrophage states aligned with mitochondrial respiration rather than aerobic glycolysis 14.

Loss of indole-producing capacity in dysbiotic microbiota therefore diminishes AhR-dependent transcriptional restraint, shifting macrophages toward inflammatory amplification 26. In this context, tryptophan metabolism operates as a metabolic rheostat that tunes the threshold for switch stabilization.

#### Pathobiont-Derived Toxins and Epitranscriptomic Disruption

In contrast to metabolite-mediated regulatory signals, certain pathobionts introduce disruptive inputs that actively destabilize macrophage metabolic equilibrium. Enterotoxigenic Bacteroides fragilis (ETBF), for example, produces B. fragilis toxin (BFT), which suppresses METTL3 expression and alters m⁶A RNA modification patterns in macrophages 27.

Such epitranscriptomic perturbations enhance translation of inflammatory mediators and glycolytic enzymes, thereby reinforcing pro-inflammatory switch configurations. Unlike SCFA or indole signaling, which bias macrophages toward oxidative states, toxin-mediated inputs directly promote glycolytic and inflammasome-driven stabilization.

These observations illustrate that microbial sensing is not inherently regulatory; it may encode either stabilizing or destabilizing information. Within the MOMS framework, pathobiont-derived signals function as high-gain amplifiers that accelerate inflammatory fate locking.

### Logical Architecture of Microbiota-Macrophage Crosstalk in the MOMS Framework

Rather than representing a simple ligand-receptor interaction, microbiota-macrophage crosstalk can be conceptualized as a structured information-processing circuit within the MOMS hierarchy. In this view, microbial communities do not merely trigger macrophage activation; they encode metabolic information that is interpreted, integrated, and stabilized into durable intracellular states.

#### Signal Encoding: Microbial Communities as Metabolic Information Sources

The intestinal microbiota generates a dynamic repertoire of metabolites, toxins, and structural ligands. These molecular products collectively encode the ecological state of the gut environment, including nutrient availability, microbial competition, and barrier integrity 28.

In homeostasis, high levels of SCFAs and indole derivatives signal a fiber-rich, symbiotic ecosystem. In dysbiosis, depletion of regulatory metabolites and enrichment of pathobiont-derived toxins signal ecological instability. These compositional shifts alter the qualitative and quantitative nature of signals perceived by intestinal macrophages 29. Thus, microbial dysbiosis should be viewed not merely as a compositional imbalance, but as a distortion of metabolic information fidelity entering the Sense layer.

#### Signal Integration: Convergence of PRR and Metabolic Sensing Pathways

Within macrophages, microbial ligands and metabolites are not processed independently. Pattern-recognition receptors (e.g., TLRs, NOD-like receptors) activate canonical inflammatory signaling cascades, while metabolite-sensing pathways (e.g., GPCRs, AhR, FXR, AMPK, mTOR) modulate cellular metabolism and chromatin configuration 30-32.

These pathways converge on shared transcriptional regulators such as HIF-1α, NF-κB, and PPARγ 33, 34. Importantly, metabolic sensors influence the amplitude and persistence of PRR-induced responses. For example, glycolytic flux enhances inflammasome activation, whereas oxidative metabolism restrains excessive cytokine production 35.

This convergence transforms transient microbial exposure into a graded intracellular response, preparing the ground for switch stabilization.

#### State Stabilization and Feedback Amplification

A defining feature of microbiota-macrophage crosstalk in chronic IBD is the persistence of inflammatory macrophage states despite fluctuations in microbial stimuli. This persistence arises when integrated sensory inputs engage the metabolic-epigenetic circuitry described in the Switch layer 36, 37.

Once glycolytic-inflammasome-m⁶A amplification loops are activated, macrophages may enter semi-stable attractor states that resist spontaneous reversal 34, 38. These stabilized states then reshape the intestinal ecosystem by promoting epithelial damage, neutrophil recruitment, and altered microbial niches.

In this feedback loop, macrophage command outputs further distort microbial composition, reinforcing dysbiosis and sustaining inflammatory input signals. Microbiota-macrophage crosstalk therefore operates as a bidirectional, self-reinforcing circuit rather than a linear activation cascade.

### Metabolite-Sensing Receptors in Intestinal Macrophages

The “Sense” layer of the MOMS framework is grounded in a defined set of metabolite-sensing receptors expressed by intestinal macrophages. These receptors translate luminal metabolic cues into intracellular signaling programs and can be broadly categorized into membrane-bound G protein-coupled receptors (GPCRs), ligand-activated nuclear receptors, and intracellular metabolic sensors.

G protein-coupled receptors (GPCRs). Short-chain fatty acids (SCFAs) are sensed by GPR43 (FFAR2), GPR41 (FFAR3), and GPR109A, which are expressed in intestinal immune cells including macrophages 12, 13, 39. Activation of these receptors modulates cAMP signaling, MAPK pathways, and inflammatory cytokine production.

Ligand-activated nuclear receptors. Tryptophan-derived indoles activate the aryl hydrocarbon receptor (AhR) 14, 26, which directly regulates transcriptional programs related to barrier integrity and immune tolerance. Bile acids engage nuclear receptors such as farnesoid X receptor (FXR) and peroxisome proliferator-activated receptor γ (PPARγ) 20, 40, linking microbial bile acid metabolism to mitochondrial function and inflammatory polarization. Liver X receptors (LXRs) further integrate lipid-derived signals with macrophage cholesterol metabolism and inflammatory tone 41.

Intracellular metabolic sensors. Beyond surface and nuclear receptors, intracellular metabolic checkpoints—including AMP-activated protein kinase (AMPK), mechanistic target of rapamycin (mTOR), hypoxia-inducible factor 1α (HIF-1α), and sirtuins (e.g., SIRT1)—act as signal integrators that couple nutrient availability to transcriptional and epigenetic regulation 38, 42, 43. These sensors bridge environmental metabolite exposure with stabilization of macrophage activation states.

Together, these receptor systems provide the molecular substrate for the Sense layer, ensuring that microbial and dietary metabolites are translated into structured intracellular decision programs within the MOMS hierarchy.

### Nutrient Competition in the Inflamed Intestinal Microenvironment

While microbial metabolites provide instructive signals to intestinal macrophages, the inflamed intestinal niche is additionally shaped by intense competition for metabolic resources. Inflammatory bowel disease is characterized not only by altered metabolite composition but also by profound redistribution of essential nutrients such as glucose, glutamine, and fatty acids among immune cells, epithelial cells, and microbial populations. This competitive landscape represents an underappreciated regulatory layer within the Sense phase of the MOMS framework.

#### Glucose Competition and Glycolytic Bias

During active intestinal inflammation, activated macrophages, infiltrating neutrophils, Th17 cells, and proliferating epithelial cells all exhibit increased glycolytic demand 44. Elevated expression of glucose transporters (e.g., GLUT1) and stabilization of HIF-1α enhance glucose uptake and aerobic glycolysis across multiple cell types 45.

Such competition results in localized glucose depletion and lactate accumulation within inflamed mucosal regions. In macrophages, limited glucose availability paradoxically reinforces glycolytic dependency through HIF-1α-mediated transcriptional amplification and succinate accumulation, further promoting NLRP3 inflammasome activation 34.

From the perspective of the MOMS framework, glucose competition does not merely reflect metabolic stress—it biases macrophages toward a glycolytic switch configuration that stabilizes inflammatory attractor states.

#### Glutamine Allocation and Epithelial-Immune Tradeoffs

Glutamine serves as a critical substrate for both rapidly proliferating epithelial stem cells and activated immune cells 46, 47. In the inflamed intestine, glutamine consumption by infiltrating immune populations may restrict availability for epithelial regeneration.

Macrophages utilize glutamine for anaplerotic replenishment of the tricarboxylic acid cycle and for redox balancing through NADPH production. Altered glutamine distribution may therefore influence mitochondrial integrity and reactive oxygen species production, modulating inflammasome sensitivity 48, 49.

Competition for glutamine thus introduces a metabolic tradeoff between immune amplification and tissue repair, directly linking nutrient allocation to downstream command outcomes.

#### Microbial Nutrient Scavenging and Ecological Distortion

Pathogenic and opportunistic bacteria further reshape nutrient availability by consuming host-derived metabolites and producing alternative metabolic byproducts 28. Dysbiotic communities may preferentially deplete short-chain fatty acids or divert tryptophan metabolism away from AhR-activating indole derivatives 14. Such microbial scavenging distorts the metabolic signals perceived by macrophages, reducing regulatory input while amplifying pro-inflammatory cues 50. This ecological distortion strengthens the probability of switch-level stabilization in inflammatory configurations.

Within the MOMS hierarchy, nutrient competition acts as a sensory amplifier rather than a downstream consequence of inflammation. By constraining metabolic flexibility and reshaping substrate availability, competition biases the intracellular integration logic described in the Switch layer. Thus, intestinal inflammation should be conceptualized not only as cytokine-driven pathology but also as a resource-driven ecological imbalance that programs macrophage fate through metabolic constraint.

## Metabolic and Epigenetic Reprogramming as the Central Switch in the MOMS framework

**Figure 2:**
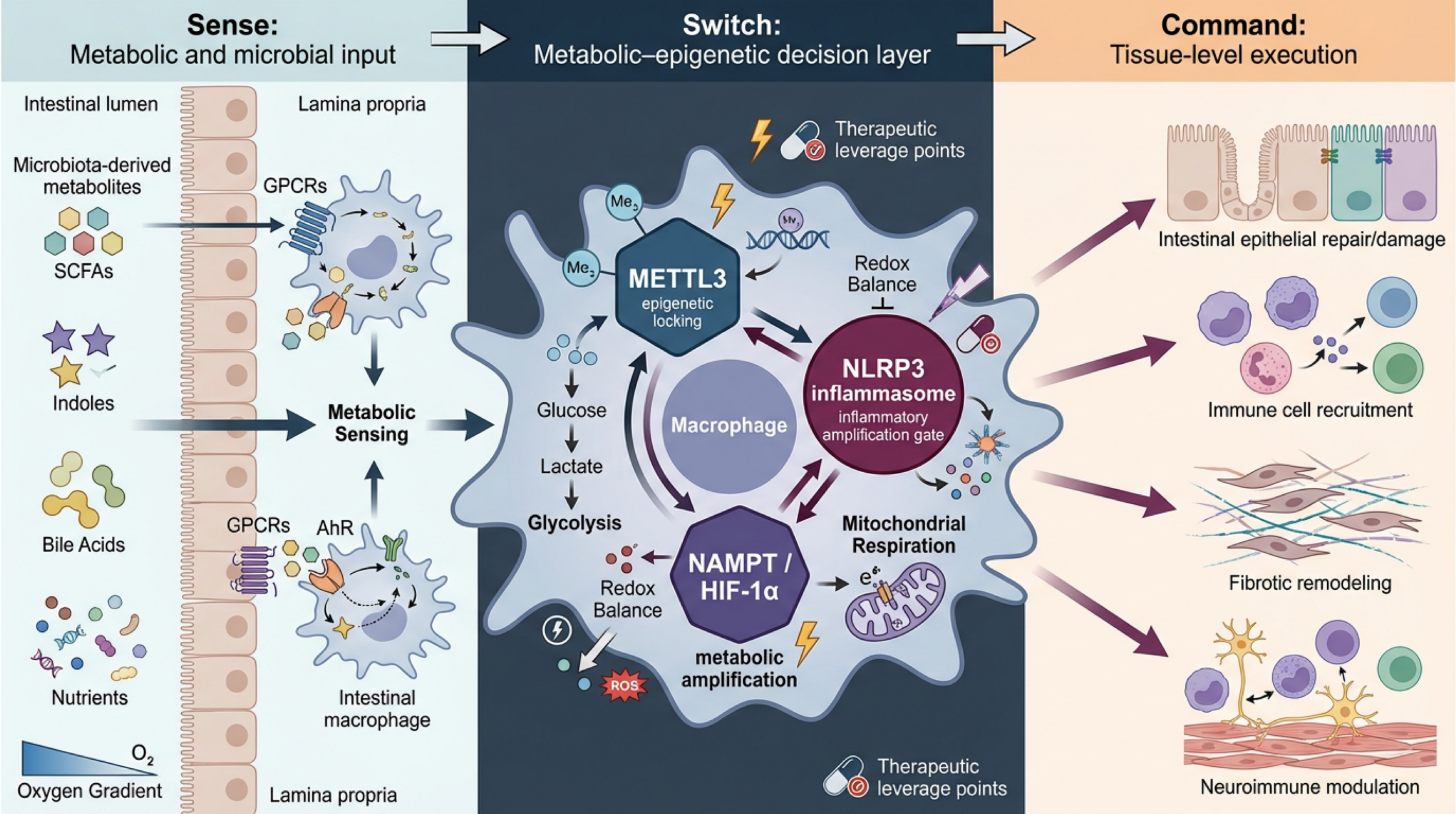
** The Macrophage-Orchestrated Metabolic Sensor (MOMS) framework in inflammatory bowel disease.** Schematic illustration of the Macrophage-Orchestrated Metabolic Sensor (MOMS) framework, which conceptualizes intestinal macrophages as hierarchical decision-making hubs that integrate environmental information to determine disease outcomes in inflammatory bowel disease (IBD). In the Sense layer, intestinal macrophages continuously detect microbiota-derived metabolites (e.g., short-chain fatty acids, indole derivatives, bile acids) and host-derived metabolic cues through dedicated receptors and intracellular sensing pathways. These inputs converge on the Switch layer, where coordinated metabolic and epigenetic reprogramming establishes durable intracellular states. Key regulatory nodes, including METTL3-mediated m⁶A RNA modification, NLRP3 inflammasome activation, and NAMPT/HIF-1α-dependent metabolic amplification, function as programmable switches that translate transient signals into stable macrophage phenotypes. Reprogrammed macrophages subsequently execute context-dependent Command functions, directing epithelial repair or damage, immune cell recruitment, fibrotic remodeling, and neuroimmune modulation. Directional information flow and feedback coupling across the Sense-Switch-Command layers enable macrophages to actively orchestrate inflammatory persistence or resolution, highlighting actionable intervention points for precision macrophage-directed therapies in IBD.

Upon sensing a complex array of microbial- and host-derived metabolites, intestinal macrophages undergo profound intracellular reprogramming that dictates whether the immune response resolves or escalates 51. This dynamic decision-making process forms the “Switch” layer within the Macrophage-Orchestrated Metabolic Sensor (MOMS) framework. The macrophage's functional fate hinges on coordinated alterations in metabolic pathways, mitochondrial integrity, and epigenetic landscapes, which collectively translate environmental cues into specific transcriptional outputs 43. These integrated mechanisms allow macrophages to function as pivotal sensors of both microbial dysbiosis and host metabolic shifts, thereby influencing the direction and magnitude of immune responses in the gut 52. This hierarchical Sense-Switch-Command architecture of the MOMS framework is schematically illustrated in **Figure 2**.

**Figure 2** should be interpreted not as a linear cascade but as a hierarchical decision structure. The Sense layer represents distributed environmental input modules, including metabolite receptors and intracellular metabolic sensors. These inputs converge on the Switch layer, which functions as a stabilization hub integrating metabolic flux, redox state, and epitranscriptomic modification into semi-stable attractor states. The Command layer translates these stabilized intracellular configurations into coordinated tissue-level outputs. Bidirectional arrows denote feedback coupling, whereby command-level inflammation further reshapes sensory input through microbiota alteration and epithelial barrier disruption, reinforcing the decision circuit.

### Metabolic Reprogramming of Macrophages in Inflammatory Disease

While macrophage metabolic reprogramming is often described in terms of shifts between glycolysis and oxidative phosphorylation, an equally important dimension lies in the signaling properties of metabolites themselves 38. Within the MOMS framework, metabolites should not be viewed solely as bioenergetic substrates, but as informational regulators that directly modulate epigenetic configuration, transcription factor activity, and inflammatory signaling pathways 34.

Short-chain fatty acids (SCFAs), particularly butyrate and propionate, function as histone deacetylase (HDAC) inhibitors, thereby reshaping chromatin accessibility and stabilizing anti-inflammatory transcriptional programs 53. Lactate, traditionally considered a glycolytic by-product, has been shown to induce histone lactylation, influencing gene expression patterns associated with macrophage polarization 54. Tryptophan-derived indole metabolites activate the aryl hydrocarbon receptor (AhR), a ligand-dependent transcription factor that directly reprograms macrophage gene expression 14. Similarly, bile acids engage nuclear receptors such as FXR and PPARγ, linking luminal metabolite composition to transcriptional control of mitochondrial function and inflammatory tone 20, 40.

Beyond extracellular sensing, intracellular metabolites—including succinate, citrate, α-ketoglutarate, and NAD⁺—act as cofactors or competitive substrates for dioxygenases, demethylases, and sirtuins, thereby integrating metabolic flux with epigenetic modification 43, 55. These mechanisms illustrate that metabolites serve as direct modulators of macrophage decision circuitry.

Thus, within the MOMS architecture, metabolites operate at both the Sense and Switch layers: they are detected as environmental signals and simultaneously function as intracellular regulators that stabilize macrophage fate through metabolic-epigenetic coupling.

#### Glycolytic Shift and Inflammatory Amplification

In inflammatory bowel disease (IBD), intestinal macrophages undergo a characteristic metabolic transition that favors aerobic glycolysis over mitochondrial oxidative phosphorylation (OXPHOS) 56, 57. This metabolic shift resembles the Warburg effect described in cancer cells 58, and supports the rapid generation of ATP and biosynthetic intermediates required for inflammatory mediator production 59.

However, this glycolytic reprogramming extends beyond energy supply. Enhanced glycolysis is accompanied by mitochondrial dysfunction, increased production of reactive oxygen species (ROS), and accumulation of metabolic intermediates that promote inflammasome activation 60. These changes amplify the secretion of pro-inflammatory cytokines such as IL-1β and TNF-α, thereby reinforcing inflammatory circuits within the intestinal mucosa 61.

Importantly, glycolysis-associated transcription factors, including HIF-1α, further stabilize inflammatory gene expression programs. Together, these processes establish a metabolically reinforced inflammatory phenotype that sustains macrophage-driven pathology in chronic IBD.

#### Oxidative Metabolism and Tissue Repair

In contrast, macrophages with anti-inflammatory and tissue-reparative functions typically rely on mitochondrial respiration and fatty acid oxidation 62. These metabolic pathways enable macrophages to maintain redox balance, promote resolution of inflammation 63.

Oxidative macrophages are typically associated with the production of IL-10, TGF-β, and growth factors that facilitate epithelial regeneration and mucosal healing 64. Maintenance of mitochondrial integrity and fatty acid oxidation capacity is therefore closely linked to the emergence of reparative macrophage subsets.

In the inflamed gut, persistent inflammatory signals suppress oxidative programs and limit the generation of these restorative macrophages 65. The imbalance between glycolytic and oxidative states thus represents a central metabolic divergence that shapes macrophage function and disease progression in IBD.

### Epigenetic Regulation of Macrophage Polarization

Recent studies highlight that metabolic transitions in macrophages are tightly linked to epigenetic regulation, suggesting a dual-layered mechanism of macrophage programming 66. A key player in this process is RNA methylation, particularly the modification of mRNA by N^6^-methyladenosine (m^6^A), catalyzed by the methyltransferase METTL3^67^. In IBD-associated macrophages, upregulation of METTL3 enhances the translation of glycolytic enzymes and pro-inflammatory cytokines, effectively locking macrophages into a state of heightened glycolysis and inflammation 68. This modification acts as a molecular switch that not only fine-tunes metabolic processes but also dictates the macrophage's polarization toward a pro-inflammatory phenotype 69.

Pharmacological or genetic inhibition of METTL3 has been shown to reverse this inflammatory bias, restoring anti-inflammatory programs and alleviating intestinal inflammation in experimental models 70. These findings underscore the role of RNA modifications as active determinants of macrophage fate, which adds an additional layer of control to macrophage polarization in IBD 71. The ability to manipulate this epigenetic regulation presents an exciting therapeutic opportunity for modulating immune responses in chronic inflammatory diseases like IBD 68.

While METTL3 functions as the primary m⁶A “writer” driving pro-inflammatory macrophage programs, the dynamic regulation of m⁶A modification also depends on “eraser” enzymes such as FTO and ALKBH5. Emerging evidence suggests that FTO-mediated demethylation can attenuate inflammatory transcript stability, whereas ALKBH5 may influence metabolic gene expression in a context-dependent manner.

The balance between m⁶A writers and erasers therefore determines the persistence or reversibility of macrophage polarization states. Future studies should clarify whether dysregulation of this dynamic equilibrium contributes to fate stabilization in chronic IBD.

### Additional Molecular Switches and Redox Regulation

While RNA methylation represents a critical epigenetic switch in macrophage polarization, other molecular regulators play essential roles in integrating metabolic inputs with transcriptional outputs 72. Histone deacetylases (HDACs) are key players in modifying chromatin structure in response to nutrient availability, which in turn influences gene expression programs 73. Redox-sensitive regulators such as Atox1 further connect intracellular redox states to transcriptional control, shaping macrophage responses to metabolic changes 74. Additionally, innate immune signaling complexes, such as the cGAS-STING pathway, link mitochondrial stress to the production of type I interferons, which have been implicated in chronic inflammatory responses 75.

At the heart of these regulatory networks is the NLRP3 inflammasome, a key molecular hub in macrophages that regulates the secretion of IL-1β and IL-18, two potent pro-inflammatory cytokines 76. Chronic activation of the NLRP3 inflammasome drives the sustained inflammatory response observed in IBD, promoting epithelial damage and the recruitment of immune cells that exacerbate tissue injury 77. Therefore, the interplay between metabolic shifts and inflammasome activation is a crucial determinant of macrophage-driven inflammation 78.

### Integrated Metabolic-Epigenetic Circuitry as a Fate-Locking Module

Although metabolic and epigenetic regulators are often studied individually, accumulating evidence indicates that they function as an interconnected network 79. In intestinal macrophages, changes in glycolytic flux, mitochondrial stress, redox balance, and m⁶A RNA modification influence each other. These interactions reinforce inflammatory transcriptional programs and make them more resistant to reversal. For example, increased glycolysis enhances mitochondrial ROS production, which promotes NLRP3 inflammasome activation 80. In parallel, METTL3-mediated m⁶A modification increases the translation of key metabolic and inflammatory genes, further sustaining glycolytic and cytokine-producing programs 81.

Enhanced glycolytic flux increases mitochondrial stress and reactive oxygen species (ROS) production, which potentiate NLRP3 inflammasome activation 82. In parallel, stabilization of HIF-1α under inflammatory or hypoxic conditions promotes transcription of glycolytic enzymes such as HK2 and PKM2, further amplifying glycolytic metabolism 83. Notably, METTL3-mediated m⁶A modification enhances the translation efficiency of key metabolic and inflammatory transcripts, including PKM2, HIF1A, TNF, and IL1B, thereby reinforcing both metabolic and cytokine-producing programs 68. PKM2 itself can translocate to the nucleus and cooperate with HIF-1α to sustain transcription of glycolytic and inflammatory genes, creating a feed-forward amplification loop 84.

Simultaneously, inflammasome-derived IL-1β signaling further stabilizes HIF-1α and sustains glycolytic reprogramming, linking innate immune activation to metabolic persistence 85. These reciprocal interactions establish a self-reinforcing network in which metabolic rewiring, epigenetic modification, and inflammatory signaling are dynamically coupled.

Conceptually, this integrated circuitry functions as a fate-locking module within the MOMS framework. Rather than representing parallel pathways, metabolic enzymes, epitranscriptomic writers, and inflammasome components collectively generate a semi-stable “attractor state” that resists spontaneous reversal even when upstream microbial or environmental stimuli fluctuate. This systems-level organization explains the durability of pathogenic macrophage states observed in chronic IBD and provides a mechanistic basis for targeting central nodes, such as METTL3 or NLRP3, to destabilize the inflammatory circuit and restore reparative programs.

The integrated metabolic-epigenetic circuitry described above gives rise to several falsifiable predictions. If macrophage fate is stabilized through glycolytic-m^6^A-inflammasome amplification loops, then patients exhibiting high expression of METTL3, HIF1A, PKM2, and NLRP3 in intestinal macrophage subsets should demonstrate greater resistance to conventional anti-cytokine therapies. Conversely, targeted disruption of these switch nodes—through pharmacologic inhibition or genetic modulation—should induce measurable shifts toward oxidative metabolism and reparative transcriptional profiles, even in the presence of persistent inflammatory stimuli. Longitudinal single-cell analyses before and after therapeutic intervention could directly test whether destabilization of switch circuits predicts durable remission.

## Intestinal Macrophages in Disease: From Inflammatory Bowel Disease to Cancer

**Figure 3:**
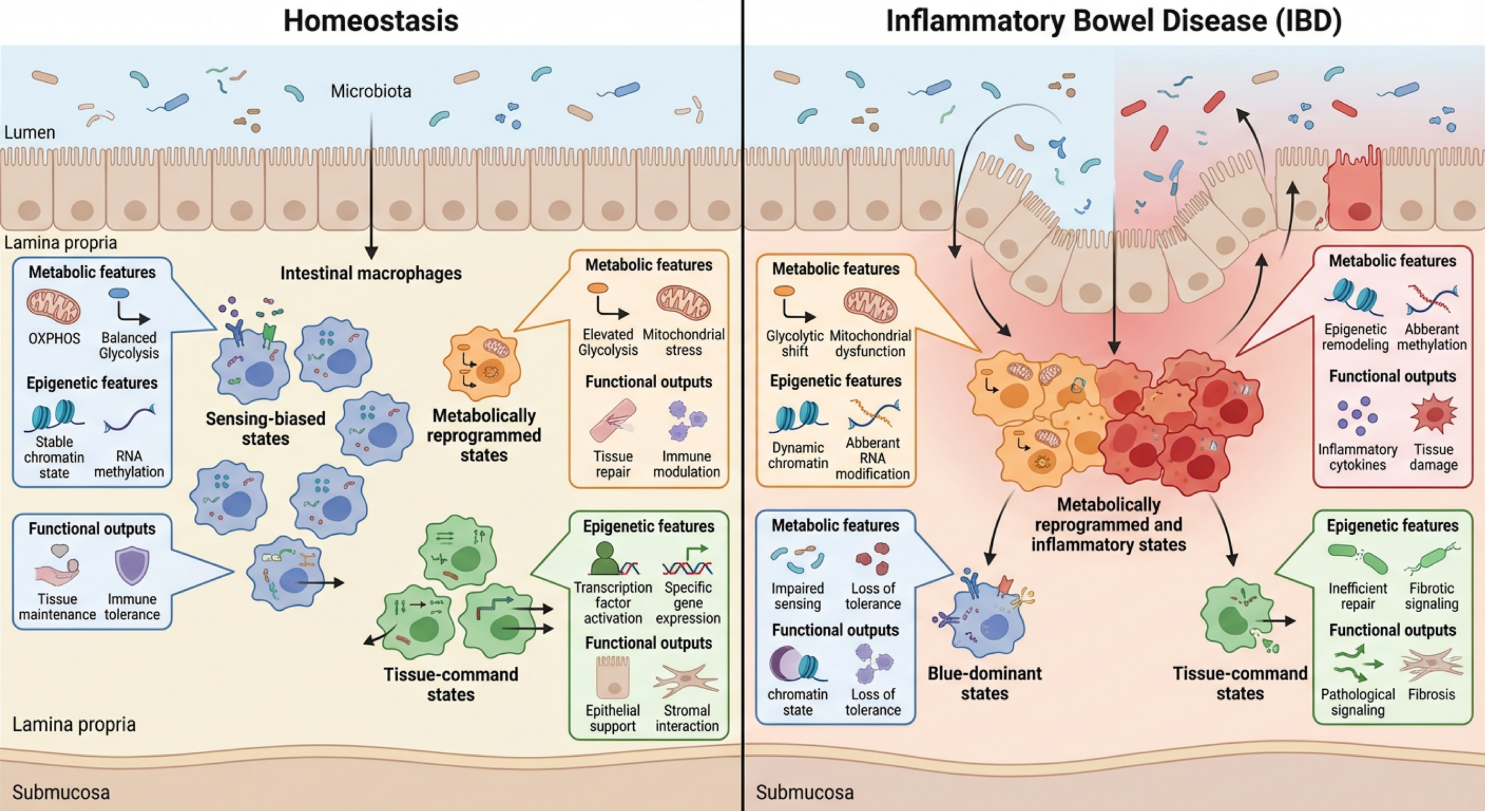
** Spatial and functional heterogeneity of intestinal macrophage states across health and disease.** Two-dimensional schematic illustrating how intestinal macrophage populations occupy distinct metabolic and functional states within the gut microenvironment. In homeostasis, macrophages are spatially distributed along the lamina propria and exhibit balanced sensing, metabolic, and reparative programs. In inflammatory bowel disease, macrophages undergo spatial redistribution and state transitions characterized by enhanced metabolic reprogramming, epigenetic remodeling, and inflammatory output. Insets highlight representative metabolic features, regulatory layers, and tissue-level consequences, illustrating how macrophage state heterogeneity underpins disease persistence and tissue remodeling.

The culmination of macrophage sensing and switching processes is manifested in the “Command” phase of the MOMS framework. As illustrated in **Figure 3**, macrophage metabolic states and functional outputs are spatially reorganized across intestinal compartments during disease progression, shaping epithelial integrity, immune recruitment, and fibrotic remodeling. In this stage, reprogrammed intestinal macrophages act as integrative decision-making hubs, converting their metabolic and epigenetic states into molecular instructions that remodel the intestinal ecosystem. These “command” signals shape epithelial turnover, immune cell composition, vascular tone, and stromal architecture—ultimately determining whether tissue homeostasis is restored or inflammation persists. Thus, macrophages not only interpret environmental information but also exert executive control over the intestinal microenvironment through precisely coordinated cytokine, chemokine, and growth factor networks.

**Figure 3** emphasizes that macrophage decision states are not uniformly distributed but spatially organized across the intestinal landscape. In homeostasis, oxidative and regulatory macrophages predominate along the lamina propria, supporting epithelial renewal. In IBD, glycolytic and inflammasome-primed subsets expand and redistribute toward ulcerated or fibrotic regions. The figure therefore visualizes how switch-level stabilization manifests as spatially distinct command outputs, linking intracellular metabolic configuration to anatomical remodeling patterns.

Crucially, the spectrum and magnitude of macrophage command outputs are directly dictated by their underlying metabolic-epigenetic configuration 38. Stabilization of HIF-1α-dependent glycolytic programs enhance transcription of chemokines such as CCL2 and CXCL8 through promoter occupancy and cooperation with nuclear PKM2, thereby sustaining monocyte and neutrophil recruitment. Concurrently, inflammasome activation amplifies IL-1β signaling, reinforcing epithelial damage and inflammatory circuit persistence 44, 86. In contrast, macrophages maintaining oxidative phosphorylation and fatty acid oxidation preferentially produce IL-10, TGF-β, and Wnt ligands, promoting epithelial regeneration and immune resolution 87. These mechanistic linkages establish a causal bridge between intracellular switch states and tissue-level command functions, reinforcing the hierarchical logic of the MOMS framework.

Although the MOMS framework positions macrophages as central decision hubs, their command functions are inseparable from reciprocal interactions with other immune populations 88. CD4⁺ T cells, particularly Th1 and Th17 subsets, provide cytokine feedback (e.g., IFN-γ, IL-17) that reinforces glycolytic and inflammasome-driven macrophage programs 89. Conversely, regulatory T cells can attenuate macrophage activation through IL-10 and CTLA-4-dependent mechanisms 90. Neutrophils recruited by macrophage-derived chemokines further amplify tissue injury through reactive oxygen species and protease release, thereby reshaping the sensory landscape encountered by macrophages 91. Fibroblasts and stromal cells also participate in bidirectional metabolic crosstalk, influencing macrophage redox balance and extracellular matrix remodeling 92.

Thus, macrophages should not be viewed as solitary drivers of pathology, but rather as nodal integrators within a distributed immune network. Their decision states both influence and are influenced by adaptive and innate immune circuits, forming feedback loops that may vary in dominance across disease phenotypes.

### Commanding Epithelial Renewal and Barrier Function

Among the most critical targets of macrophage-derived signals is the intestinal epithelium. The epithelial layer is continuously renewed by Lgr5⁺ intestinal stem cells (ISCs) residing at the crypt base, whose proliferation and differentiation are tightly regulated by macrophage-secreted mediators 93, 94. Pro-inflammatory macrophages, abundant in active IBD lesions, secrete IL-6, TNF-α, and IL-1β, which not only impair ISC proliferation but also disrupt the Wnt/β-catenin and Notch signaling axes that maintain epithelial stemness 95, 96. This disruption leads to niche collapse, epithelial apoptosis, and the formation of deep mucosal ulcers—a hallmark of severe Crohn's disease and ulcerative colitis 97.

Conversely, reparative macrophages foster epithelial restitution through the production of IL-10, TGF-β, amphiregulin, and Wnt ligands, which stimulate crypt expansion and mucosal healing 98. Recent studies have shown that macrophage-derived Wnt1 and Wnt2b directly activate ISCs, accelerating epithelial closure following injury 93, 99. Moreover, the metabolic state of macrophages—particularly their reliance on oxidative phosphorylation and fatty acid oxidation—correlates with their capacity to promote epithelial recovery 63. These findings highlight that macrophage-derived epithelial crosstalk operates as a metabolically regulated axis of mucosal regeneration, bridging the “Switch” and “Command” layers of the MOMS framework.

### Immune Cell Recruitment and Circuit Amplification

#### Chemokine Networks and Monocyte-Neutrophil Recruitment

Intestinal macrophages exert strong control over immune cell trafficking during inflammatory bowel disease (IBD). Through the secretion of chemokines such as CCL2, CXCL1, CXCL8, and CCL20, they recruit circulating monocytes, neutrophils, and effector T cells into inflamed mucosal regions 100.

This recruitment is not a transient event. Sustained chemokine production establishes a self-amplifying inflammatory circuit in which newly recruited immune cells further release cytokines and damage-associated signals, reinforcing macrophage activation 101, 102.

As a result, macrophages act as gatekeepers of immune cell influx, shaping both the magnitude and persistence of intestinal inflammation.

#### Disease-Specific Amplification in Crohn's Disease and Ulcerative Colitis

Glycolytic, pro-inflammatory macrophages (M1-like) sustain prolonged chemokine secretion through HIF-1α-dependent transcriptional programs, while anti-inflammatory macrophages downregulate these pathways and release IL-10 and resolvins that dampen immune cell influx 103, 104.

The impact of macrophage-derived chemokines differs across IBD subtypes. In Crohn's disease, elevated CCL2 expression correlates with enhanced monocyte recruitment and granuloma formation 105, 106, These infiltrating cells contribute to transmural inflammation and fibrosis.

In ulcerative colitis, excessive CXCL8 production drives neutrophil accumulation within the lamina propria and crypt lumen, leading to crypt abscess formation and epithelial erosion 107, 108. These disease-specific patterns illustrate how macrophage-derived chemokine networks translate intracellular inflammatory states into spatially organized immune pathology.

#### Metabolic Programming Links Switch to Command

Importantly, the strength and duration of chemokine signaling are tightly regulated by macrophage metabolic configuration 35. Glycolytic macrophages, characterized by HIF-1α stabilization and enhanced PKM2 activity, sustain prolonged transcription of chemokine genes 34, 109. This metabolic state supports continuous immune cell recruitment and inflammatory amplification.

In contrast, macrophages relying on oxidative phosphorylation and fatty acid oxidation downregulate pro-inflammatory chemokine production and instead secrete IL-10 and pro-resolving mediators 110. These cells limit excessive immune infiltration and promote restoration of tissue homeostasis.

Thus, immune cell recruitment represents a direct functional output of the Switch layer described in the MOMS framework. Stabilized metabolic-epigenetic states determine whether macrophages amplify inflammatory circuits or facilitate their resolution.

### Fibrosis, Stromal Remodeling, and Neuromodulatory Crosstalk

Beyond their roles in epithelial and immune regulation, macrophages command the structural remodeling of the intestinal wall through fibrotic and neuroimmune pathways 111, 112. In chronic inflammation, macrophage-derived TGF-β, PDGF, and matrix metalloproteinases (MMPs) drive fibroblast activation, myofibroblast differentiation, and extracellular matrix deposition 113-115. This contributes to collagen accumulation, intestinal wall thickening, and ultimately fibrotic stricture formation—especially in Crohn's disease. Interestingly, the macrophage metabolic profile also dictates fibrotic outcomes: glycolytic macrophages favor fibrogenesis, whereas oxidative macrophages limit collagen deposition and promote matrix resolution 63, 116.

Moreover, macrophage-neuron interactions introduce an additional layer of control over gut physiology. Through the release of neuroactive molecules such as prostaglandin E2, nitric oxide, and neurotrophins, macrophages modulate enteric neuron excitability, thereby influencing gut motility, visceral hypersensitivity, and pain perception 112, 117, 118. This emerging neuroimmune dialogue positions macrophages as key intermediaries connecting immune inflammation to functional gastrointestinal symptoms, revealing that their command extends beyond immune regulation to the sensory and neuromuscular domains of the gut.

Collectively, these downstream actions position intestinal macrophages as central command units that dictate both pathological persistence and therapeutic recovery in IBD. Their ability to simultaneously modulate epithelial repair, immune recruitment, fibrosis, and neuromodulation underscores their hierarchical role in intestinal decision-making. By reframing these processes within the MOMS framework, it becomes evident that therapeutic strategies must go beyond suppressing inflammation—they must reprogram the macrophage command logic toward restoration and equilibrium 119.

At the command level, the MOMS framework predicts that distinct macrophage metabolic states will spatially correspond to defined tissue-level outputs. For example, glycolytic and inflammasome-primed macrophage clusters should colocalize with zones of epithelial erosion, neutrophil infiltration, and fibrotic remodeling, whereas oxidative and IL-10-producing macrophages should align with regions of crypt regeneration and barrier restoration 87, 120. Spatial transcriptomics and multiplex imaging in human IBD samples provide an avenue to directly validate these associations 121. Furthermore, selective reprogramming of macrophage switch states should produce coordinated alterations across epithelial, immune, and stromal compartments, reflecting the hierarchical nature of macrophage command functions.

Targeting key molecular determinants that bridge the “Switch” and “Command” phases—such as metabolic checkpoints, m^6^A modifiers, or inflammasome regulators—could recalibrate macrophage output without compromising host defense 70. In doing so, future therapies may achieve precise immunologic balance: resolving chronic inflammation while preserving the gut's regenerative and protective functions.

## Therapeutic Targeting Within the MOMS Framework

The Macrophage-Orchestrated Metabolic Sensor (MOMS) model not only provides a conceptual understanding of intestinal immune dysregulation in IBD, but also reveals a series of actionable intervention points. By stratifying therapeutic strategies according to the Sense-Switch-Command architecture, a rational and mechanism-based roadmap for macrophage-directed therapy emerges. This paradigm moves beyond symptomatic immunosuppression and instead aims to reprogram macrophage fate at critical regulatory nodes. Based on the MOMS framework, therapeutic strategies can be systematically organized according to the macrophage decision layer they target, as summarized in ***Table 1***. Importantly, these therapeutic strategies differ fundamentally in the decision layer they target within the MOMS hierarchy. Interventions at the Sense layer recalibrate environmental input signals; Switch-layer therapies attempt to destabilize intracellular fate circuits; Command-level strategies intercept downstream outputs without necessarily modifying macrophage identity. Distinguishing these layers clarifies both the strengths and limitations of existing and emerging treatments.

### Targeting the Sense Layer: Restoring Metabolic and Microbial Inputs

Interventions at the sensory level of the MOMS framework aim to normalize the metabolic and microbial landscapes perceived by intestinal macrophages. Rather than directly suppressing inflammation, these strategies focus on recalibrating upstream cues—the biochemical signals that dictate macrophage fate—thereby influencing the entire cascade of immune and epithelial responses that follow. By reconstructing a symbiotic metabolic environment, such interventions seek to restore the physiological dialogue between the microbiota, epithelial cells, and macrophages that underpins mucosal homeostasis. From the MOMS perspective, sensory-layer interventions offer systems-level recalibration but may lack specificity and durability if switch circuits remain stabilized. Thus, microbiota restoration alone may be insufficient in patients with entrenched metabolic-epigenetic fate locking.

#### Reinstating Microbial-Derived Metabolites

Among microbial metabolites, short-chain fatty acids (SCFAs)—notably butyrate, propionate, and acetate—play a dominant role in shaping macrophage behavior 122. SCFAs, generated through bacterial fermentation of dietary fibers, exert potent anti-inflammatory effects via G-protein-coupled receptor 43 (GPR43) activation and histone deacetylase (HDAC) inhibition 123, 124. These dual actions converge to promote an anti-inflammatory, IL-10-producing macrophage phenotype characterized by enhanced mitochondrial respiration and reduced glycolytic flux 104, 122. Butyrate, in particular, has been shown to stabilize HIF-1α under hypoxic mucosal conditions, thereby enhancing epithelial barrier integrity and limiting bacterial translocation—two processes that are often disrupted in IBD 125, 126.

Therapeutic approaches to increase luminal SCFA levels have expanded rapidly. Prebiotic supplementation (e.g., inulin-type fructans, resistant starch), engineered probiotics capable of sustained butyrate release, and encapsulated SCFA formulations designed for distal colonic delivery have all demonstrated potential to indirectly reprogram macrophage function 126-128. Notably, animal studies reveal that restoring butyrate availability can attenuate colitis severity by rebalancing macrophage metabolism and reducing inflammasome activation 129, 130. The integration of such strategies into clinical practice represents a promising avenue for microbiota-informed immunotherapy, acting not through broad immune suppression but through targeted restoration of sensory input fidelity.

#### Tryptophan Metabolism and AhR-Dependent Regulation

Beyond SCFAs, tryptophan-derived indole metabolites constitute another crucial microbial signal that influences macrophage programming. Metabolites such as indole-3-aldehyde, indole-3-propionic acid, and kynurenine engage the aryl hydrocarbon receptor (AhR) on intestinal macrophages, modulating transcriptional networks involved in mucosal healing, oxidative stress regulation, and immune tolerance 131, 132. Activation of AhR enhances the expression of IL-22 and regenerating islet-derived proteins (Reg3γ) in neighboring epithelial cells, reinforcing barrier defense while constraining inflammatory macrophage polarization 133.

Supplementation with tryptophan precursors, AhR agonists, or engineered microbial consortia that restore indole metabolite biosynthesis has been shown to protect against colitis in experimental models 134, 135. Mechanistically, these interventions promote a shift from glycolytic to oxidative macrophage metabolism, concomitant with decreased production of TNF-α and IL-1β 131, 136. The resulting macrophage phenotype resembles the homeostatic populations found in healthy mucosa, capable of clearing pathogens without triggering excessive inflammation.

#### Sensory Recalibration as a Systems-Level Strategy

In the context of the MOMS framework, interventions targeting the sensory layer extend beyond simple microbiome modification. They function as information recalibrators, correcting the quality and intensity of metabolic signals entering macrophages. By reestablishing a balanced metabolic dialogue between the microbiota and host immune system, these strategies have the potential to realign downstream switching and command phases, ultimately restoring tissue equilibrium 124, 137.

This systems-level approach reframes gut immunotherapy: rather than treating macrophages as static effectors, it views them as adaptive interpreters of environmental information 116. Thus, manipulating sensory inputs—through diet, microbial engineering, or targeted metabolite delivery—represents a precision strategy to reprogram macrophage behavior at its origin, offering a foundation for sustainable remission in inflammatory bowel disease 127, 128.

### Targeting the Switch Layer: Reprogramming Macrophage Fate at the Molecular Level

The “Switch” phase represents the most powerful and direct opportunity for therapeutic intervention within the MOMS framework. At this critical juncture, the macrophage's fate—whether pro-inflammatory or reparative—is dictated by coordinated networks of metabolic flux, mitochondrial fitness, and epigenetic plasticity. Therapeutic modulation of this internal circuitry offers the potential to recalibrate macrophage behavior upstream of chronic inflammation, thus intercepting disease progression before irreversible tissue damage ensues. Advances in small-molecule inhibitors, RNA-targeted therapeutics, and nanomedicine delivery systems now enable precise intervention at this intracellular level, marking a paradigm shift from symptom suppression to macrophage reprogramming. Switch-targeted therapies hold the greatest theoretical potential for durable remission, yet they face challenges in delivery specificity and safety, given the systemic importance of metabolic and epigenetic regulators.

#### METTL3 and RNA Methylation as Master Switches

One exemplary and mechanistically validated target is METTL3, the catalytic core of the m^6^A RNA methyltransferase complex 67, 138. Elevated METTL3 expression in intestinal macrophages correlates strongly with enhanced glycolytic metabolism, stabilization of pro-inflammatory transcripts (e.g., HIF1A, IL6, TNF), and suppression of anti-inflammatory gene networks 68, 70. Mechanistically, METTL3-mediated m^6^A modification promotes the translation of glycolytic enzymes such as PKM2 and ENO1, reinforcing a metabolic lock that sustains inflammatory activation 67, 68.

Pharmacological inhibition or siRNA-mediated silencing of METTL3 disrupts this pathogenic feedback loop, restoring oxidative phosphorylation and reactivating reparative signaling pathways such as PPARγ and AMPK 139. In murine colitis models, METTL3 blockade attenuates inflammatory cytokine release, improves epithelial barrier integrity, and accelerates mucosal healing 140. These findings position METTL3 as a central epigenetic “master switch” in the MOMS framework—an intracellular gatekeeper whose manipulation can pivot macrophage identity from destructive to restorative.

Beyond METTL3, additional m^6^A writers (e.g., METTL14), erasers (e.g., FTO, ALKBH5), and readers (e.g., YTHDF1/2) have also been implicated in macrophage polarization 141-146, suggesting that a broader m^6^A-targeted therapeutic strategy may allow multilayered control over macrophage transcriptional output. Emerging RNA-targeted platforms, including antisense oligonucleotides and CRISPR-based epitranscriptomic editing, are expected to refine this level of precision control in the near future 147.

#### Inflammasome Suppression: Resetting the Command Logic

Another pivotal regulatory node within the Switch phase is the NLRP3 inflammasome, which integrates metabolic stress signals with innate immune activation. Overactivation of NLRP3 sustains the production of IL-1β and IL-18, thereby amplifying epithelial injury and perpetuating the inflammatory milieu characteristic of IBD 148-150. The inflammasome thus functions not only as an effector platform but also as a metabolic-immunologic amplifier linking mitochondrial dysfunction, ROS accumulation, and lysosomal damage to chronic inflammation 151, 152.

Pharmacological inhibition of NLRP3 using small molecules such as MCC950, or downstream blockade of caspase-1 activity, has shown protective effects in experimental colitis models 153-155. These agents effectively dampen IL-1β signaling, reduce macrophage infiltration, and preserve epithelial architecture. Notably, inflammasome inhibition does not ablate macrophage antimicrobial functions 156, indicating that such interventions can selectively “reset” the macrophage's command logic without compromising host defense. In this way, NLRP3 represents a tractable target through which the pathological momentum of the Switch phase can be reversed.

#### Redox and NAD⁺ Metabolism as Metabolic Levers

Beyond epigenetic and inflammasome regulation, redox-sensitive proteins and NAD⁺ metabolic enzymes constitute additional control nodes in macrophage reprogramming. Proteins such as Atox1 respond to intracellular oxidative stress by modulating copper-dependent redox signaling and transcriptional responses, thus linking environmental stress to macrophage activation states 74. Similarly, enzymes involved in NAD⁺ metabolism, such as NAMPT (nicotinamide phosphoribosyltransferase) and SIRT1, play central roles in balancing energy metabolism and inflammatory tone 157, 158.

Enhancement of NAD⁺ availability through precursors (e.g., nicotinamide riboside or nicotinamide mononucleotide) or inhibition of NAMPT-driven NAD⁺ depletion has been shown to restore mitochondrial respiration, reduce ROS accumulation, and suppress pro-inflammatory transcriptional programs in intestinal macrophages 159, 160. These interventions realign macrophage metabolism toward an oxidative, tissue-protective phenotype—highlighting the therapeutic value of redox and NAD⁺ pathways as metabolic “dials” capable of fine-tuning macrophage fate.

#### Toward Precision Macrophage Reprogramming

Collectively, these switch-level targets—spanning RNA methylation, inflammasome signaling, and redox metabolism—constitute a precision-oriented therapeutic platform aimed at intercepting pathogenic macrophage programming at its origin. By targeting intracellular nodes that determine polarization rather than downstream inflammatory mediators, such strategies offer the promise of durable immune recalibration 70, 157.

Integration of these molecular insights with emerging nanocarrier-based drug delivery systems could further enhance specificity, allowing the selective delivery of METTL3 or NLRP3 inhibitors directly to intestinal macrophages 161, 162. This convergence of epigenetic editing, metabolic rewiring, and nanomedicine represents a transformative therapeutic direction: one that seeks not merely to suppress inflammation, but to redefine the immune logic governing macrophage behavior within the MOMS framework. However, the intracellular localization and systemic importance of these molecular switches present substantial formulation and safety challenges, underscoring the necessity for macrophage-selective delivery platforms discussed below.

### Targeting the Command Layer: Blocking Pathological Outputs

The final tier of intervention within the MOMS framework focuses on intercepting the harmful downstream commands issued by pathogenic macrophage populations. In this “Command” phase, macrophages translate their internal metabolic and epigenetic states into cytokine, chemokine, and growth factor outputs that sculpt the tissue environment. Therapeutic efforts at this level therefore aim to neutralize or rechannel these molecular orders, preventing further damage to the intestinal epithelium and stromal compartments while allowing reparative processes to prevail. Command-layer blockade remains clinically effective but primarily addresses symptomatic output rather than upstream decision logic, potentially explaining relapse following treatment discontinuation.

#### Cytokine Neutralization and Immune Circuit Disruption

Neutralization of pro-inflammatory cytokines, including TNF-α, IL-1β, and IL-6, remains the cornerstone of current IBD management. Monoclonal antibodies such as infliximab (anti-TNF-α), ustekinumab (anti-IL-12/23p40), and anti-IL-6 receptor antagonists have demonstrated clinical efficacy by disrupting cytokine-driven inflammatory circuits that perpetuate epithelial injury 163-165. However, from the perspective of the MOMS framework, these interventions act as output-level correctors—they intercept pathological macrophage commands without addressing the upstream logic that generates them 166-168. Consequently, their efficacy often wanes over time, and patients may experience disease relapse upon treatment discontinuation.

Recent translational studies suggest that combining cytokine blockade with macrophage reprogramming strategies (e.g., METTL3 or NLRP3 inhibition) may achieve more stable disease remission 70, 153. Such combination therapy targets both the source and the signal, simultaneously silencing inflammatory outputs and recalibrating macrophage input-output relationships. Moreover, selectively inhibiting context-specific cytokine networks—for instance, blocking IL-1β in fibrosis-dominant Crohn's phenotypes or IL-6 in ulcerative colitis—could refine therapeutic precision and minimize systemic immunosuppression 169, 170.

#### Chemokine and Fibrotic Signaling Modulation

Beyond cytokines, macrophage-derived chemokines and growth factors serve as critical effectors of immune recruitment and tissue remodeling 171. The CCL2/CCR2 axis exemplifies this, orchestrating the continuous influx of monocytes into inflamed mucosa and sustaining the inflammatory circuit 172, 173. Pharmacological inhibitors of CCR2, such as cenicriviroc, have shown promise in experimental colitis by reducing macrophage accumulation and dampening tissue injury 174. Similarly, blockade of CXCL8/CXCR2 signaling limits neutrophil-driven epithelial destruction and may synergize with anti-TNF therapy in refractory cases 175.

Macrophage-derived growth factors—including TGF-β, platelet-derived growth factor (PDGF), and connective tissue growth factor (CTGF)—mediate fibroblast activation and collagen deposition, driving intestinal fibrosis and stricture formation 176. Targeting these downstream effectors, either via TGF-β receptor inhibitors, anti-PDGF antibodies, or small-molecule tyrosine kinase blockers, offers a means to intercept fibrogenic command pathways 177-180. Importantly, the MOMS framework suggests that these fibrotic outputs are not isolated phenomena but reflections of persistent pro-inflammatory command logic; thus, reprogramming upstream macrophage states may prevent fibrosis more effectively than late-stage antifibrotic therapy alone 63.

#### Integrated Output Modulation and Systems-Level Recalibration

From a systems biology perspective, the “Command” phase represents the interface between macrophage-intrinsic programming and tissue-level homeostasis. Modulating this phase requires not only blocking harmful signals but also restoring constructive communication between macrophages, epithelial cells, and the stromal niche 181. Novel biologics and engineered cytokines that mimic reparative signals—such as IL-10 analogs, amphiregulin, and resolvin derivatives—are being investigated to actively promote mucosal restitution 182-186.

Furthermore, advances in nanomedicine and targeted delivery systems now enable spatially confined intervention at the command level. Nanocarriers loaded with cytokine-neutralizing agents or siRNAs can be designed to preferentially accumulate in inflamed intestinal segments, ensuring localized modulation of macrophage outputs while minimizing systemic exposure 187-192. These strategies complement upstream “Switch” interventions by forming a closed-loop therapeutic network, where internal macrophage reprogramming and output correction reinforce each other to restore homeostatic equilibrium 193.

#### The MOMS Perspective: From Suppression to Reprogramming

Within the MOMS paradigm, therapies acting at the “Command” phase should no longer be viewed merely as inflammatory suppressants, but as circuit modulators—agents that reshape the downstream expression of macrophage-derived decisions 194. Their integration with metabolic and epigenetic reprogramming at the “Switch” phase establishes a multi-tiered therapeutic logic: Rewire the macrophage's internal state (Switch). Recode its communication signals (Command). Restore tissue-level harmony and immune homeostasis.

By aligning downstream blockade with upstream reprogramming, this integrated approach may achieve what isolated cytokine therapies cannot—durable remission through macrophage logic restoration, rather than transient suppression of inflammation.

### Macrophage-Targeted Delivery Systems: Translating the MOMS Framework into Precision Therapy

A central implication of the MOMS framework is that effective therapeutic intervention requires selective modulation of intracellular “Switch” nodes within intestinal macrophages. Targets such as METTL3, NLRP3, and redox-sensitive metabolic regulators are not merely extracellular mediators but intracellular determinants of fate commitment. Systemic inhibition of these pathways, however, risks disrupting macrophage-mediated host defense and tissue homeostasis in distant organs. Therefore, spatially confined and cell-specific delivery is not an auxiliary consideration—it is an essential enabling technology for translating MOMS-inspired interventions into clinical reality.

Macrophage-targeted delivery systems thus represent the engineering arm of the MOMS framework. By leveraging macrophage-specific surface receptors, inflammatory microenvironment cues, and innate phagocytic properties, these platforms enable selective reprogramming of pathogenic macrophage states while minimizing systemic immunosuppression. Conceptually, such delivery systems function as therapeutic gatekeepers that ensure intervention occurs precisely at the Switch layer within disease-relevant macrophage populations.

#### Active Targeting: Ligand-Receptor-Guided Macrophage Selectivity

Active targeting strategies exploit surface receptor signatures characteristic of intestinal macrophage subsets. Among these, the mannose receptor (CD206) has been widely utilized, given its enriched expression on alternatively activated or tissue-resident macrophages in inflamed mucosa 195. Mannose-modified nanoparticles constructed from PLGA 196, liposomes 197, or chitosan 198 derivatives enhance receptor-mediated endocytosis and significantly improve macrophage-specific uptake in preclinical colitis models.

Beyond mannose-CD206 interactions, additional ligand-guided systems have been developed targeting folate receptors, scavenger receptors, Fc receptors, and chemokine receptors such as CCR2^199^, ^200^. These approaches exploit intrinsic macrophage identity rather than inflammatory environment alone, allowing selective accumulation within macrophage-dominant lesions. Such receptor-guided precision is particularly valuable for delivering siRNA, mRNA, or small-molecule inhibitors directed at intracellular Switch regulators such as METTL3 or NLRP3^201^, ^202^.

Conceptually, active targeting aligns with the MOMS principle of cellular specificity: interventions are directed toward the decision-making hubs themselves rather than broadly suppressing downstream inflammatory mediators.

#### Microenvironment-Responsive Platforms: Exploiting the Inflammatory Niche

In addition to receptor-guided targeting, a complementary strategy involves engineering nanocarriers that respond to pathological features of the inflamed intestinal microenvironment. Chronic IBD lesions are characterized by reduced pH, elevated reactive oxygen species (ROS), enhanced protease activity, and high local concentrations of inflammatory cytokines. Microenvironment-responsive systems exploit these biochemical cues to trigger site-specific drug release 203.

pH-sensitive polymeric vesicles, mucoadhesive nanoassemblies, and ROS-responsive nanoparticles have demonstrated preferential retention and controlled payload release in inflamed colonic segments. Redox-sensitive disulfide linkers, for example, enable cargo liberation within macrophage phagosomes enriched in glutathione 204. Similarly, ROS-responsive scaffolds release anti-inflammatory agents upon encountering oxidative stress, thereby synchronizing therapeutic activation with inflammatory intensity 205.

This strategy reflects a spatial logic consistent with the MOMS framework: therapeutic modulation is confined to regions where the Sense layer is pathologically amplified, ensuring that Switch-level reprogramming occurs selectively within disease-relevant macrophage populations.

#### Bio-Inspired and Living Carriers: Harnessing Physiological Communication Pathways

A third and increasingly sophisticated category comprises bio-inspired or living carriers that exploit endogenous cell-cell communication systems. Exosome-mimetic vesicles, engineered bacterial ghosts, and immune cell-activated nanoassemblies (ICANs) leverage natural biological tropisms to enhance macrophage selectivity and biocompatibility 206-208.

These systems offer several conceptual advantages. First, they mirror physiological intercellular signaling pathways, increasing integration within the intestinal ecosystem. Second, they may allow multi-functional design—combining drug delivery, imaging capability, and microenvironment responsiveness within a single platform. Third, bio-inspired systems reduce immunogenicity and may better navigate mucosal barriers compared with synthetic nanoparticles.

By co-opting biological transport logic, these carriers extend the MOMS principle beyond molecular reprogramming to ecosystem-level engineering.

#### Smart Nanocarriers as Synthetic Analogues of the MOMS Framework

The most advanced macrophage-targeted systems increasingly mirror the hierarchical architecture of the MOMS framework itself. Immune Cell-Activated Nanoassemblies (ICANs), for example, remain inert under physiological conditions but become activated upon sensing inflammatory cues such as elevated TNF-α, excessive ROS, or acidic pH 209-211. Upon detection of these disease-specific inputs (Sense), the nanocarrier undergoes structural transformation or cargo release (Switch), subsequently delivering payloads—such as siRNA, small-molecule inhibitors, or metabolic modulators—that recalibrate macrophage output programs (Command).

In this manner, next-generation smart nanocarriers function as synthetic analogues of the MOMS logic: they sense pathological microenvironmental signals, execute an internal switching event, and deliver precise commands to reprogram macrophage fate. This convergence between biological decision-making and engineered therapeutic systems underscores the translational power of the MOMS framework. Delivery platforms are thus not passive vehicles but programmable extensions of macrophage-centered immunometabolic control.

The integration of macrophage-selective targeting with Switch-level molecular reprogramming represents a critical step toward precision immunometabolic therapy. By coupling intracellular circuit destabilization with spatially confined activation, such systems offer the potential to achieve durable remission while preserving systemic immune competence.

### Safety Considerations and Theoretical Risk-Benefit Comparison

While targeting the metabolic “Switch” layer offers a mechanistically appealing strategy for durable macrophage reprogramming, it also raises important safety considerations. Unlike cytokine blockade, which primarily neutralizes extracellular effector signals, modulation of intracellular regulators such as METTL3 or NLRP3 may exert broader systemic effects due to their roles in host defense and tissue homeostasis.

Potential risks of METTL3 modulation. METTL3-mediated m^6^A RNA methylation participates in hematopoietic differentiation, antiviral responses, and epithelial regeneration 212. Systemic inhibition could theoretically impair immune cell turnover, compromise antiviral defense, or affect rapidly proliferating tissues 213. Moreover, prolonged alteration of RNA methylation dynamics may influence oncogenic or tumor-suppressive pathways, raising concerns regarding long-term malignancy risk 214.

Potential risks of NLRP3 inhibition. NLRP3 inflammasome activity is critical for sensing bacterial and fungal pathogens 215. Sustained suppression may increase susceptibility to opportunistic infections, similar in principle to anti-TNF-associated infection risk. Additionally, inflammasome signaling contributes to tissue repair in certain contexts; its chronic inhibition could interfere with mucosal healing dynamics 216.

Compared to anti-TNF therapy, which acts at the level of inflammatory cytokine neutralization, targeting the Switch layer represents a more upstream intervention 217. Theoretical advantages include the possibility of reprogramming macrophage states rather than transiently suppressing effector cytokines, potentially reducing relapse driven by metabolic memory. However, this upstream positioning also implies broader biological integration, increasing the importance of cell-specific delivery strategies and dosing precision.

In this regard, macrophage-targeted nanoparticle delivery systems, gut-restricted formulations, or transient modulation approaches may mitigate systemic exposure. Importantly, the MOMS framework does not advocate indiscriminate systemic inhibition of METTL3 or NLRP3, but rather highlights these nodes as mechanistic entry points for precision immunometabolic intervention.

Ultimately, the safety profile of Switch-targeting strategies will depend on tissue specificity, duration of intervention, and patient stratification—factors that must be rigorously evaluated in translational studies.

## Challenges and Future Directions

While the MOMS framework provides a structured model for macrophage-centered disease orchestration in IBD, several biological and translational challenges must be addressed before its full clinical implementation.

### Inter-Patient Heterogeneity and Stratification

Human IBD exhibits profound inter-patient heterogeneity at genetic, microbial, metabolic, and immunologic levels. Single-cell transcriptomic studies demonstrate that intestinal macrophage subsets vary not only in abundance but also in metabolic configuration across individuals and disease stages 120. Consequently, the dominant drivers of macrophage dysfunction—whether sensory deficits, switch-level stabilization, or command-phase amplification—may differ between patients.

Without robust stratification strategies, macrophage-directed therapies risk variable efficacy. Future research should focus on identifying clinically feasible biomarkers that reflect macrophage metabolic-epigenetic states. Multi-omic integration of biopsy-derived transcriptomics, spatial profiling, and fecal metabolomics may enable classification of patients according to dominant macrophage switch configurations.

### Temporal Plasticity and Fate Stabilization

Although the MOMS framework emphasizes semi-stable metabolic-epigenetic states, macrophage phenotypes remain dynamically influenced by the inflammatory microenvironment. Cytokine gradients, hypoxia, microbial perturbations, and metabolic stress continuously reshape macrophage programming 218.

A key unanswered question is the temporal window during which macrophage states remain reversible. Early intervention before stabilization of inflammatory circuits may yield greater therapeutic benefit than late-stage intervention. Longitudinal sampling and time-resolved single-cell analyses will be essential to define when macrophage switch destabilization is most achievable 219.

### Safety and Delivery Specificity

Many proposed switch-level targets—including METTL3, NLRP3, NAMPT, and redox regulators—are broadly expressed across tissues. Systemic modulation of these pathways may compromise host defense or disrupt physiological immune functions in other organs 214, 216, 220.

Although macrophage-targeted delivery systems offer a promising solution, ensuring selective and safe intracellular modulation in human patients remains challenging. Issues such as off-target accumulation, immunogenicity, scalability of nanocarriers, and long-term safety require rigorous evaluation in translational studies.

### Human Validation Beyond Experimental Models

Most mechanistic evidence supporting macrophage metabolic switching derives from murine colitis models 221. However, these systems often represent acute or chemically induced inflammation, whereas human IBD is chronic, relapsing, and influenced by environmental and therapeutic variables 222.

Human intestinal macrophages display greater transcriptional diversity and spatial complexity than murine counterparts 219. Furthermore, prior exposure to biologics, corticosteroids, or immunomodulators may alter macrophage metabolic states in patient samples. Validation of the MOMS framework in human biopsy-derived macrophages, organoid-immune co-culture systems, and longitudinal clinical cohorts is therefore essential.

### Conceptual Boundaries of a Macrophage-Centered Model

Although macrophages function as key integrators of intestinal immunity, they are not the sole drivers of disease. In certain genetic contexts, adaptive immune dysregulation—such as IL-10 receptor deficiency or regulatory T cell defects—may precede macrophage stabilization 223, 224. In these settings, macrophages may amplify rather than initiate inflammatory cascades.

Moreover, single-cell studies reveal hybrid macrophage populations that co-express inflammatory and reparative signatures, suggesting that switch states may exist along a spectrum rather than as discrete attractors 225. Determining whether these hybrid states represent transitional intermediates or stable configurations remains an open question.

Finally, systemic metabolic conditions—including obesity, insulin resistance, and micronutrient deficiencies—may influence macrophage behavior through endocrine pathways not fully incorporated into the current framework 226. Expanding the MOMS framework to integrate organism-level metabolism represents an important future direction.

### Toward Precision Macrophage Reprogramming

Addressing these challenges will require integration of computational modeling, multi-omic patient profiling, and targeted delivery technologies 227. By combining mechanistic validation with clinically actionable biomarkers, future studies may enable mechanism-guided therapy selection based on dominant macrophage metabolic configurations.

Rather than replacing existing biologics or microbiota-based interventions, macrophage reprogramming strategies may complement current treatments, potentially reducing required dosages and improving durability of remission 228.

Taken together, these challenges delineate a translational roadmap for transforming macrophage-centered metabolic decision theory into precision immunotherapy for IBD and related inflammatory diseases.

## Spatial Metabolomics as a Critical Validation Layer for the MOMS Framework

The MOMS framework posits that intestinal macrophage decision states are spatially organized and metabolically constrained within the inflamed tissue microenvironment. While single-cell transcriptomics and spatial transcriptomics have revealed marked heterogeneity in macrophage gene expression across intestinal compartments 120, these approaches do not directly resolve the spatial distribution of metabolites that shape macrophage sensing and switch stabilization.

Spatial metabolomics—enabled by imaging mass spectrometry, matrix-assisted laser desorption/ionization (MALDI)-based profiling, and emerging high-resolution metabolite mapping technologies—offers a complementary dimension capable of reconstructing metabolite gradients across the crypt-luminal axis and within ulcerated or fibrotic lesions 229.

### Mapping Metabolite Gradients Across Intestinal Compartments

In healthy intestine, gradients of short-chain fatty acids, oxygen tension, bile acids, and amino acids are spatially organized along the mucosal surface and lamina propria 230. In IBD, these gradients are disrupted by epithelial erosion, vascular remodeling, and immune infiltration 231.

Spatial metabolomic mapping may reveal localized depletion of SCFAs in ulcerated regions, accumulation of lactate in neutrophil-rich zones, or altered tryptophan metabolite distribution in areas of dysbiosis. Such information would directly test the hypothesis that sensory input fidelity varies across anatomical microdomains.

### Linking Metabolite Topology to Macrophage State Distribution

A central prediction of the MOMS framework is that macrophage metabolic states should colocalize with specific metabolite environments. Glycolytic and inflammasome-primed macrophage clusters are expected to occupy regions characterized by glucose competition, hypoxia, and succinate accumulation 34, 45. In contrast, oxidative and IL-10-producing macrophages may preferentially localize to zones enriched in regulatory metabolites such as butyrate and indole derivatives 232.

Integrating spatial metabolomics with multiplex immunofluorescence or spatial transcriptomics would allow direct correlation between metabolite topology and macrophage switch configuration. Such spatial co-mapping could distinguish whether inflammatory macrophage stabilization is driven primarily by intrinsic epigenetic locking or by persistent local metabolic constraint.

### Spatially Resolved Validation of Command Outputs

Beyond sensing and switching, spatial metabolomics may illuminate how macrophage command functions reshape the metabolic microenvironment. For example, areas of intense IL-1β production may exhibit localized shifts in redox state or lipid oxidation products 80. Fibrotic regions dominated by macrophage-derived TGF-β may display altered extracellular matrix-associated metabolic signatures 92.

Thus, spatial metabolomics does not merely complement transcriptomic profiling; it provides a mechanistic bridge connecting metabolic sensing, intracellular stabilization, and tissue-level remodeling.

### Toward Integrated Multi-Omic Spatial Modeling

The full validation of the MOMS framework will likely require integration of spatial metabolomics, single-cell transcriptomics, proteomics, and microbiome profiling within the same tissue specimens 233. Computational reconstruction of macrophage decision states within defined metabolic niches could reveal patient-specific patterns of sensory distortion or switch stabilization.

Such multi-omic spatial integration would transform MOMS from a conceptual framework into a quantitatively testable systems model, enabling identification of spatially constrained therapeutic windows and microdomain-specific intervention strategies.

## Conclusion

Inflammatory bowel disease arises from complex interactions among the intestinal microbiota, host metabolism, and immune regulation. In this review, we propose the Macrophage-Orchestrated Metabolic Sensor (MOMS) framework, which reframes intestinal macrophages as programmable metabolic decision hubs that integrate environmental signals to determine inflammatory persistence or tissue repair. Within this hierarchical Sense-Switch-Command architecture, macrophages first detect microbial and host-derived metabolic cues, then stabilize intracellular programs through metabolic and epigenetic circuitry, and ultimately execute tissue-level commands that shape epithelial renewal, immune cell recruitment, and stromal remodeling. By positioning metabolism as a causal driver rather than a passive consequence of immune activation, the MOMS framework provides a conceptual bridge linking microbial ecology, immunometabolism, and disease progression in IBD.

Importantly, the framework generates several testable predictions with direct translational relevance. First, distinct metabolic and transcriptional signatures of intestinal macrophages are expected to correlate with disease state, severity, and therapeutic responsiveness in patients with IBD. Second, targeted modulation of key molecular “switches,” including regulators such as METTL3, NLRP3, and redox-sensitive metabolic checkpoints, may redirect macrophage fate from pathogenic inflammatory programs toward reparative states. Third, precision delivery systems capable of selectively targeting intestinal macrophages—such as ligand-guided nanoparticles or bio-inspired carriers—may enable localized immune reprogramming without the burden of systemic immunosuppression.

Beyond molecular predictions, the hierarchical structure of the MOMS framework provides a foundation for systems-level modeling of macrophage decision states. Integration of multi-omic datasets—including single-cell transcriptomics, spatial metabolomics, microbiome profiling, and immune repertoire sequencing—may enable computational reconstruction of patient-specific macrophage metabolic configurations. Such approaches could facilitate mechanism-guided patient stratification, allowing clinicians to predict whether individual patients are more likely to benefit from interventions targeting sensory inputs (microbiota modulation), intracellular switch circuits (metabolic or epigenetic reprogramming), or downstream inflammatory outputs.

From a therapeutic perspective, the MOMS framework suggests that durable remission in IBD may require not only suppression of inflammatory mediators but also reprogramming of macrophage decision logic. Macrophage-directed strategies may therefore complement existing biologics, microbiota-based therapies, and small-molecule inhibitors by addressing upstream determinants of immune persistence. Importantly, such approaches aim to restore balanced macrophage signaling rather than indiscriminately suppress immune function.

Although this review focuses on inflammatory bowel disease, the conceptual implications of the MOMS framework likely extend to a broader spectrum of macrophage-driven disorders. Similar metabolic-epigenetic switching mechanisms have been implicated in tumor-associated macrophages, metabolic syndrome-related inflammation, fibrotic diseases, and neuroinflammatory conditions. By conceptualizing macrophages as programmable metabolic decision hubs, the MOMS framework may therefore provide a unifying perspective for understanding chronic immune-metabolic diseases across tissues.

Together, these insights highlight macrophage metabolic sensing and intracellular switch stabilization as central determinants of inflammatory persistence and therapeutic responsiveness. We anticipate that continued integration of multi-omic profiling, spatial analysis, and targeted delivery technologies will enable the translation of macrophage-centered metabolic decision theory into precision immunotherapy for IBD and related diseases.

## Supplementary Material

Supplementary figures.

## Figures and Tables

**Table 1:** Conceptual comparison between MOMS and existing models

Feature	Classical Immunometabolism	Microbiota-Immune Model	Cytokine Cascade Model	MOMS Framework
Role of metabolism	Downstream support	Modulatory	Irrelevant	Causal driver
State stabilization	No	No	No	Yes
Explains persistence after anti-TNF failure	Limited	No	No	Yes
Predicts spatial heterogeneity	No	Limited	No	Yes
Provides stratification logic	No	No	No	Yes

**Table 2:** Representative microbial metabolites regulating macrophage polarization in IBD

Microbial Metabolite	Primary Receptor/Pathway	Effect on Macrophage Metabolism	Functional Outcome	Reference
Butyrate (SCFA)	GPR43, GPR109A, HDAC inhibition	Enhances mitochondrial respiration; reduces glycolysis	Promotes IL-10 production and epithelial repair	11-13, 17
Propionate	GPR43	Modulates AMPK activity	Anti-inflammatory polarization	12, 18
Indole derivatives	AhR signaling	Promotes oxidative metabolism	Enhances barrier protection and immune tolerance	14, 19
Secondary bile acids	FXR, PPAR	Regulates mitochondrial function	Limits inflammasome activation	20, 21
Polyamines	mTOR and redox pathways	Modulates cytokine translation	Alters macrophage activation profile	15
