# Supplementary material for: Macrophage-Orchestrated Metabolic Sensing Drives IBD Pathogenesis: A Framework for Targeted Therapy
Source: Int J Biol Sci. 2026 May 25;22(11):5754–79. doi: 10.7150/ijbs.131531 (PMC13282748; doi:10.7150/ijbs.131531)
Supplement: Supplementary file 1 — Supplementary figures. [file ijbsv22p5754s1.pdf]

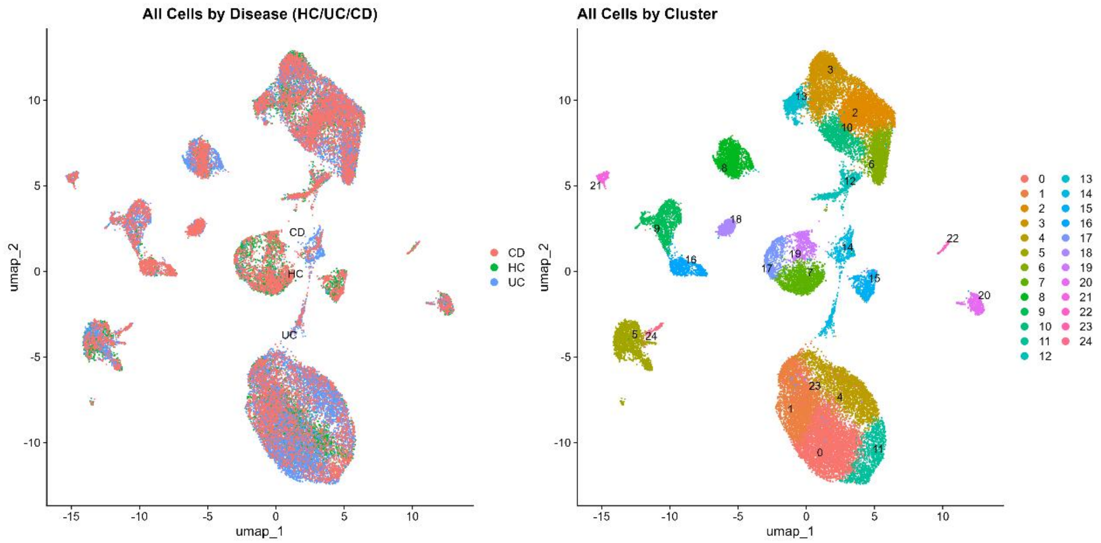

Figure S1. Global single-cell transcriptional landscape of intestinal mucosa in health and IBD

UMAP visualization of integrated scRNA-seq data from colonic biopsies of healthy controls (HC) and patients with ulcerative colitis (UC) or Crohn's disease (CD) (GSE214695). Cells are colored by disease status (left) and unsupervised clusters (right). This overview reveals pronounced compositional and transcriptional remodeling in inflamed tissues, with notable expansion of myeloid lineages, establishing the cellular context for macrophage-centric dysregulation in IBD and motivating focused interrogation of their role in the proposed MOMS framework.

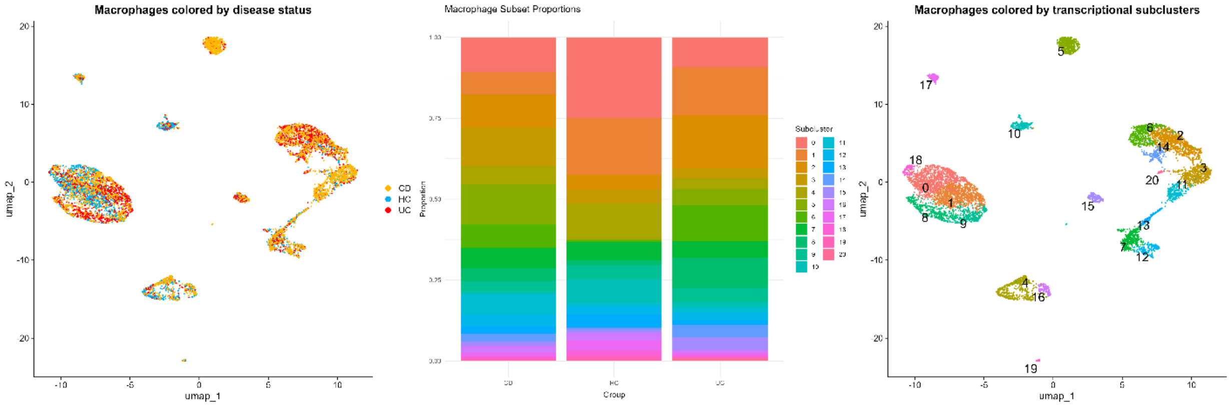

Figure S2.Disease-driven expansion of pro-inflammatory macrophage subsets in IBD

Macrophages and monocytes were extracted based on canonical myeloid marker expression and re-embedded in UMAP space. Left: cells colored by disease status (HC, UC, CD), illustrating global transcriptional divergence between healthy and inflamed states. Right: cells colored by unsupervised subclusters, delineating intrinsic functional heterogeneity. Middle: stacked bar plot quantifying subcluster proportions across disease groups. Side-by-side comparison demonstrates selective enrichment of pro-inflammatory macrophage subsets in UC and CD, providing direct single-cell evidence for inflammation-dependent polarization imbalance—a foundational element of the metabolic-orchestrated microenvironment switch (MOMS) model.

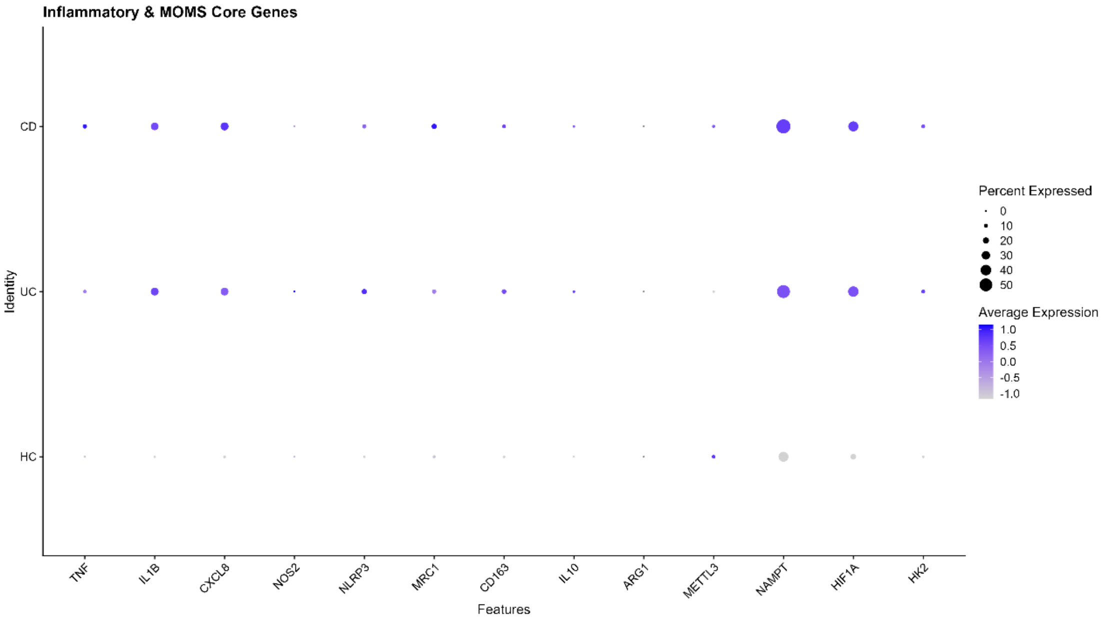

Figure S3.Differential expression of inflammatory and metabolic regulatory genes across macrophage subclusters

Dot plot showing scaled expression levels and percentage of expressing cells for selected pro-inflammatory (TNF, IL1B, CXCL8, NOS2, NLRP3), resident/anti-inflammatory (MRC1, CD163, IL10, ARG1), and MOMS core metabolic/epigenetic regulators (METTL3, NAMPT, HIF1A, HK2, PKM2) across macrophage subclusters. Disease-enriched subclusters exhibit elevated glycolytic and inflammasome-associated gene signatures, independently validating the metabolic reprogramming switch layer central to macrophage-mediated inflammatory amplification in IBD.

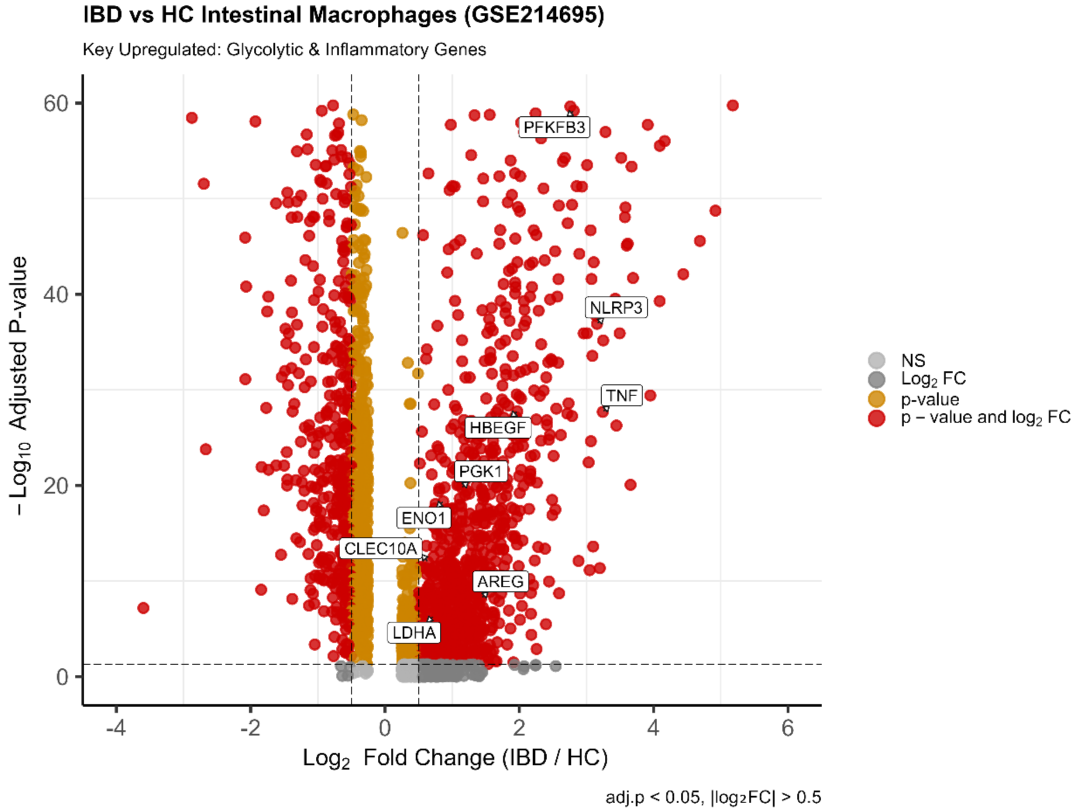

Figure S4.Glycolytic reprogramming and inflammatory activation in IBD-associated intestinal macrophages

Volcano plot (left) showing differentially expressed genes in intestinal macrophages from IBD patients (UC+CD pooled) versus healthy controls (HC) based on reanalysis of GSE214695 scRNA-seq data. Key upregulated genes related to inflammation (e.g., TNF, IL1B, NLRP3) and glycolysis (e.g., HK2, PKM2, PFKFB3, LDHA) are labeled (adj. $p < 0.05$, $|\log_2 FC| > 0.5$).

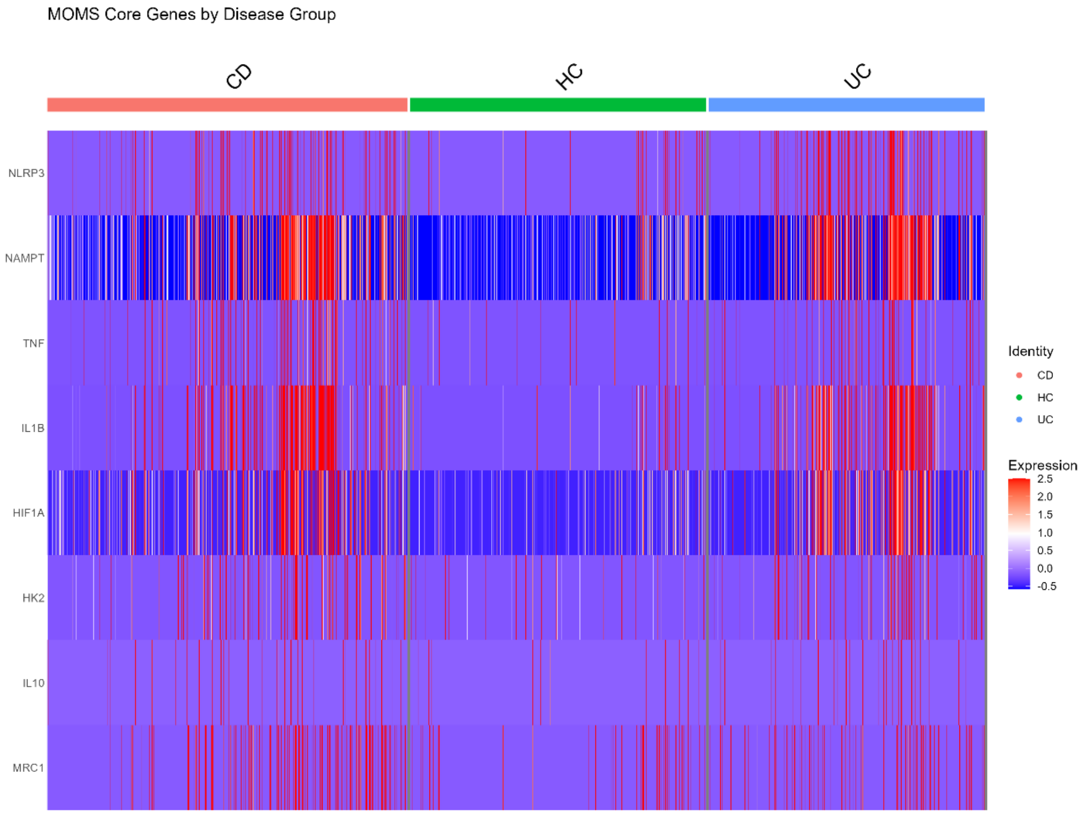

Figure S5.Upregulation of MOMS model core regulatory nodes in diseased macrophages

Heatmap depicting expression of ten pivotal MOMS regulators (METTL3, NLRP3, NAMPT, TNF, IL1B, HIF1A, HK2, PKM2, IL10, MRC1) across individual macrophages stratified by disease status. Pronounced overexpression of metabolic sensors (NAMPT, HIF1A), epigenetic modifiers (METTL3), and effector modules (NLRP3, pro-inflammatory cytokines) in UC and CD samples corroborates the interconnected metabolic–epigenetic–inflammatory axis orchestrated by intestinal macrophages, reinforcing their role as central commanders of the IBD microenvironment.
